# Systematic Literature Review Shows Gaps in Data on Global Prevalence and Birth Prevalence of Sickle Cell Disease and Sickle Cell Trait: Call for Action to Scale Up and Harmonize Data Collection

**DOI:** 10.3390/jcm12175538

**Published:** 2023-08-25

**Authors:** Raffaella Colombatti, Inga Hegemann, Morten Medici, Camilla Birkegård

**Affiliations:** 1 Clinic of Pediatric Hematology Oncology, Department of Child and Maternal Health, Azienda Ospedaliera, University of Padova, 35122 Padua, Italy; 2 Novo Nordisk Pharma AG, 8058 Zürich, Switzerland; inhm@novonordisk.com; 3 Novo Nordisk A/S, 2860 Søborg, Denmark; vmei@novonordisk.com (M.M.); acbk@novonordisk.com (C.B.)

**Keywords:** sickle cell disease, sickle cell trait, sickle cell anemia, sickle beta-thalassemia, epidemiology

## Abstract

Sickle cell disease (SCD) is an inherited monogenic disorder with high prevalence throughout sub-Saharan Africa, the Mediterranean basin, the Middle East, and India. Sources of SCD epidemiology remain scarce and fragmented. A systematic literature review (SLR) to identify peer-reviewed studies on SCD epidemiology was performed, with a search of bibliographic databases and key conference proceedings from 1 January 2010 to 25 March 2022 (congress abstracts after 2018). The SLR followed PRISMA (Preferred Reporting Items for Systematic Reviews and Meta-Analyses) guidelines. Meta-analyses, using a binomial normal random-effects model, were performed to estimate global and regional prevalence and birth prevalence. Of 1770 journal articles and 468 abstracts screened, 115 publications met the inclusion criteria. Prevalence was highest in Africa (~800/100,000), followed by the Middle East (~200/100,000) and India (~100/100,000), in contrast to ~30/100,000 in Europe. Birth prevalence was highest in Africa (~1000/100,000) and lowest in North America (~50/100,000) and Europe (~30/100,000). This SLR confirmed that sub-Saharan and North-East Africa, India, the Middle East, and the Caribbean islands are global SCD hotspots. Publications including mortality data were sparse, and no conclusions could be drawn about mortality. The identified data were limited due to gaps in the published literature for large parts of the world population; the inconsistent reporting of SCD genotypes, diagnostic criteria, and settings; and a sparsity of peer-reviewed publications from countries with assumed high prevalence. This SLR demonstrated a lack of systematic knowledge and a need to provide uniform data collection on SCD prevalence and mortality.

## 1. Introduction

Sickle cell disease (SCD) is one of the most common severe monogenic disorders in the world [1,2]. SCD is a hereditary chronic hemolytic anemia resulting from mutations in the subunit β-chain gene of hemoglobin (Hb) that cause stiff, sickle-shaped red blood cells that are involved in a complex series of adhesive events among blood cells, leading to small blood vessel obstruction, which reduces the oxygen supply to body tissues. Sickle-shaped red blood cells are prone to hemolysis, entailing at least three separate mechanisms involved in SCD pathophysiology: endothelial damage with vaso-occlusion, vasculopathy, and anemia [1,2]. Patients with SCD suffer from repeated infections, tissue hypoxia, acute and chronic pain, and multi-organ damage [1,3,4].

The acute and chronic complications of SCD, particularly vaso-occlusive crises, require frequent emergency room visits and hospitalizations [1,3]. Eventually, cumulative organ damage reduces survival compared with the general population [5]. The introduction of newborn screening programs and preventative measures have contributed to the improved life expectancy of patients with SCD, but the mortality rate in low-income regions is still high in children ≤3 years of age [6].

There are various genotypes that cause SCD, and the genotypes generally result in different severities of symptoms. Homozygosity for the sickle cell mutation in the Hb gene (HbS), known as sickle cell anemia or HbSS, is the most common and severe type of SCD [3,7]. Other types of SCD involve one HbS gene plus other mutations of the Hb gene, which lead to other altered forms of hemoglobin such as HbC [1,7]. HbSC is a common compound genotype that generally leads to a different phenotype, with fewer critical events but more retinopathy than HbSS [1]. HbS/β^+/0^-thalassemia, which is compound heterozygosity for HbS and β^0^ or β^+^ (the latter two leading to the impaired production of Hb β-chains), occurs in approximately 10% of people with SCD [3,7]. The HbS/β^+^ genotype generally results in a milder phenotype, whereas the HbS/β^0^ genotype leads to a severe form of SCD comparable to HbSS [3,7,8]. There is also a range of rarer genotypes, including regional variants, such as HbSD^Punjab^, HbSE, HbSO^Arab^, and HbS Lepore [1,7,9]. People with one HbS gene and one normal gene (HbAS or sickle cell trait, SCT) rarely have symptoms or complications, although some evidence exists of possible progressive organ damage in some individuals with HbAS [1,7].

SCD is highly prevalent throughout large areas of sub-Saharan Africa, the Mediterranean basin, the Middle East, and India, which is possibly related to the high level of protection that SCT provides against severe malaria [2]. Studies suggest that approximately 300,000 infants worldwide are born every year with SCD [2]. A study with the objectives of estimating and mapping the global distribution of neonates with heterozygous (HbAS) and homozygous (HbSS) genotypes found that frequencies of HbS (>10%) were almost exclusively encountered in sub-Saharan Africa, with additional relatively high frequencies in India [10]. In 2008, the World Health Organization estimated that the global prevalence of SCD was 2.28 cases per 1000 people [11]. A systematic literature review (SLR) of 67 papers published between January 1980 and July 2017 reported a global birth prevalence of SCD homogenous genotypes (HbSS) in children under 5 years age of 112/100,000 live births, with a birth prevalence in Africa of 1125/100,000 compared with 43/100,000 in Europe [12].

With the increased global population movement, the prevalence and disease burden of SCD are changing in various regions. However, studies estimating the current prevalence of SCD and the mortality associated with it are scarce; fragmented; and dependent on sources that are difficult to compare (e.g., administrative databases, registries, screening programs, and clinical trials), limiting our understanding of the number of contemporary patients living with SCD across specific geographies worldwide and the impact that this disease has on their life expectancy. It is important to obtain sound data on prevalence to understand the health economic burden and complications of SCD in patients with various genotypes, as it will ensure the correct assessment of healthcare needs, assist policymakers with evidence-based decision making on how to allocate funding and resources, and determine adequate prevention and screening strategies.

The objective of this study was to perform an SLR of recent publications, from 2010 to 2022, on the prevalence, birth prevalence, and mortality associated with SCD globally and regionally, and to identify any knowledge gaps.

## 2. Materials and Methods

This SLR was performed in accordance with guidelines from the National Institute for Health and Clinical Excellence (NICE) using methodologies from the Cochrane Collaboration [13,14] and Preferred Reporting Items for Systematic Reviews and Meta-Analysis (PRISMA) guidance [15,16], and the study was registered with PROSPERO (ID 321574). The results are reported according to the 2020 PRISMA statement [17].

This SLR drew on data that are readily available in the public domain; hence, it did not require ethical review and approval.

### 2.1. Search Strategy

A comprehensive literature search of bibliographic databases and proceedings from key conferences was performed to identify peer-reviewed studies reporting on SCD epidemiology from 1 January 2010 to 25 March 2022. Congress publications published before 2018 were excluded, as it was assumed they would have been published subsequently as full-text articles. The bibliographic databases searched were: Excerpta Medica Database (Embase), MEDLINE, MEDLINE Epub Ahead of Print, BIOSIS, and Current Contents. The conference abstract databases searched were: Insightmeme and Embase. The proceedings from key congresses, the European Haematology Association, and the American Society of Hematology were also searched.

The search terms included a combination of subject headings (namely, variations of thesaurus and indexing terms (for example, MESH in MEDLINE)) and ‘free-text’ terms (e.g., synonyms, acronyms and abbreviations, spelling variants, and medical terminology) related to the prevalence or birth prevalence of SCD, common SCD genotypes, and SCT, or its associated impact on mortality. Comprehensive details of the search terms for prevalence, birth prevalence, and mortality and the searches performed can be found in Table S1.

Additional full-text articles and congress publications outside of the search identified as references within references, but relevant for outcomes of interest, could be included. In addition, data from national databases could be included to cover any gaps in peer-reviewed publications.

### 2.2. Eligibility Criteria for Publications

A Population, Intervention, Comparison, Outcomes, and Study (PICOS) framework was used to formulate the inclusion/exclusion criteria for the SLR (outlined in Table S2). All publications between 1 January 2010 and 25 March 2022 published in English, French, Spanish, or other languages where an English abstract was available, were eligible for inclusion if the population studied included people with any form of SCD (including HbSS, HbSC, HbS/β^0^-thalassemia, HbS/β^+^-thalassemia, HbSD, or HbSO^Arab^) or SCT, irrespective of age (including children, adolescents, and adults); race; gender; or setting, and only if the publication had an outcome measure of interest (prevalence of SCD/SCT, birth prevalence of SCD/SCT, mortality related to SCD and its complications, or life expectancy in people with SCD/SCT).

Studies that were not deemed eligible for inclusion were animal studies, in vitro/ex vivo studies, gene/protein expression studies, or modeling studies; single-center studies (unless the center gathered data from a wider geographic region than a single town or community); and studies that reported data from a specific ethnic group within a country. Conference or meeting abstracts, meeting posters, or conference papers published before 2018 were excluded. Reviews and literature reviews; letters, case reports, editorials, comments, or editorial material; news, interviews, lectures, video-audio material, tombstone advertisements, meeting summary errata, corrections, and retracted publications were also excluded. For any studies published in more than one report, the most comprehensive and up-to-date version was used.

### 2.3. Study Selection

All identified search hits were imported into a reference management program (Endnote X9) for duplication checks. Two independent reviewers screened the titles and abstracts of records identified in the searches using the pre-defined eligibility criteria. Any discrepancies were resolved by consensus and by consulting a third reviewer as needed.

Congress abstracts and abstracts of full-text peer-reviewed publications deemed relevant during the first review step were retrieved, reviewed again, and selected for data extraction based on the eligibility criteria by the two independent reviewers, with a third reviewer to resolve any differences.

**Figure 1:**
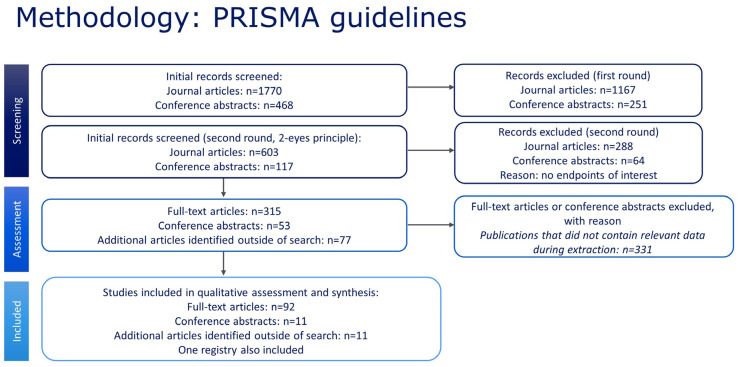
PRISMA diagram for the search strategy and selected studies.

After identifying and validating the articles to be included in the review, a full list of included studies was collated. The flow diagram of screening, assessment, and inclusion is outlined in Figure 1.

### 2.4. Study Quality

Study quality was assessed by two independent reviewers using a version of the Grading of Recommendations, Assessment, Development, and Evaluations (GRADE) framework, adapted to observational studies. In this adaptation, publication bias and dose–response gradient were not evaluated, as publication bias would be difficult to assess in the context of epidemiological studies (e.g., a comparison of published literature versus registered clinical trials), and dose–response gradients do not apply to a non-interventional setting. The risk of bias was assessed in terms of factors that allowed the true estimate to be under-/over-reported. A GRADE assessment was applied to each study, rather than outcome. Where the assessment varied between outcomes, the highest-graded outcome was used to guide the overall study assessment.

### 2.5. Data Extraction, Reporting, and Synthesis

An extraction dashboard set up in a spreadsheet program was used to capture the data. The variables with sufficient data to be included in this report were: (1) authors, year, and title of publication; (2) year(s) of data collection; (3) greater region (regions defined based on available data in current SLR); (4) country; (5) number of people with SCD; (6) total population at risk; (7) disease classification: genotype, including HbSS, HbSC, sickle β-thalassemia, and regional SCD variants, or SCT; (8) proportion male (%); (9) prevalence data: prevalence (%), number of people with genotype; (10) birth prevalence data: birth prevalence (%); (11) mortality rate: by age category; (12) mean survival: survival curves; (13) cause of mortality; and (14) study quality: very low, low, moderate, or high. Other variables were extracted but were not sufficiently comprehensive to use in further analyses. A complete list of the parameters extracted is given in Table S3.

Data on the prevalence, birth prevalence, and mortality from the included studies were synthesized by either SCD genotype (HbSS, HbSC, sickle HbS/β-thalassemia [HbS/β^0^-thalassemia, HbS/β^+^-thalassemia] or undefined if the genotype was not described or if it was mixed) or SCT, as well as by greater region (Africa, Europe, India, Middle East, North America, and South America/the Caribbean). Initially, data were intended to be reported from Asia as a region. However, this was revised to India as a region, as no data were available from other countries in Asia. Studies investigating prevalence in children <1 year of age were considered to report on birth prevalence, while data from older children, adolescents, and adults were included as a prevalence measurement.

### 2.6. Quantitative Analyses of Prevalence and Birth Prevalence

A summary estimate was determined for the prevalence and birth prevalence globally and regionally (when sufficient data were available). The summary estimates were calculated from a random-effects model that assumed a binomial distribution for the results of each individual study, with the true value drawn from a normal distribution centered on a true regional/global value (assumed to exist). This will be referred to as a binomial normal (BN) model. Inhomogeneity scores (I^2^) are reported for each summary estimate to quantify the model assumption of a shared regional/global prevalence. The calculations were made using R statistics software [18] with the metafor package [19].

The summary estimates are presented as forest plots for prevalence and birth prevalence for SCD grouped (i.e., across all SCD genotypes) by individual SCD genotypes (when sufficient data were available), and for SCT. The quantitative analyses only included studies concerning groups that represented the general population and had a total study population larger than 1000.

### 2.7. Quantitative Analyses of Mortality

Due to the heterogeneity in reporting mortality across the identified studies, data on mortality are presented as survival curves. Publications found in the SLR with information on survival rates were identified and the data extracted. When a dataset with all individuals and their censoring or death date was not available, the survival curves presented in the papers were either digitized to extract the values, or survival curves were drawn based on other mortality information (e.g., mortality rate or deaths and at-risk population). For papers reporting survival rates for intervals, the survival curves reflected this by plotting the corresponding drop in the middle of the interval. If only deaths and at-risk population were reported, the survival probability was estimated by the Kaplan–Meier estimator.

In addition to the survival curves for SCD identified through the SLR, the equivalent curve for the general population was acquired from either the Human Mortality Database (HMD) [20] or the Human Life-Table Database (HLD) [21]. Notably, not all SCD populations had a corresponding general population from the same country and years. In these cases, a survival curve for the general population from another country in the same region, or the same country and another year, was used for comparison.

## 3. Results

Of 1770 journal articles and 468 abstracts screened, 115 publications met the inclusion criteria for the SLR (Figure 1). Of these, 23 included data on SCD prevalence and 23 on SCT prevalence, 50 included data on the birth prevalence of SCD, and 43 on the birth prevalence of SCT. Thirty-three publications included some aspect of data on mortality or life expectancy.

### 3.1. Qualitative Data Analysis

Key data on prevalence and birth prevalence for SCD/SCT are synthesized in Tables S4–S7 in the Supplementary Materials. Mortality data were extracted from the text, and the identified causes of death among people with SCD are shown in Table S8 in the Supplementary Materials. Data extracted from the text, tables, and figures were used to generate survival curves.

### 3.2. Quantitative Meta-Analysis of Prevalence and Birth Prevalence

**Figure 2:**
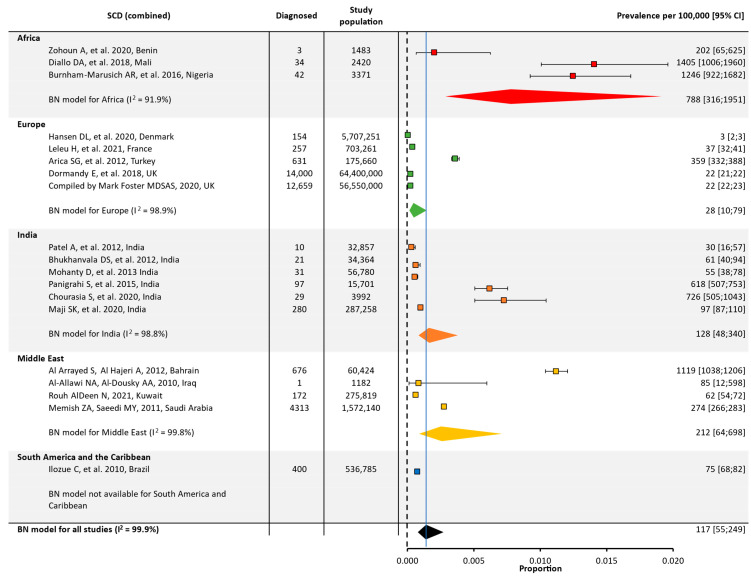
Global and regional prevalence ^a^ of SCD in Africa [22,23,24], Europe [25,26,27,28,29], India [30,31,32,33,34,35], the Middle East [36,37,38,39], and South America/the Caribbean [40]. ^a^ Within each region, the prevalence was estimated using a binomial normal model, which assumed a binomial distribution for the individual studies with a mean value drawn from a normal distribution for a regional/global value. The prevalence for each reference was determined from the log odds. A summary estimate was determined for each region with >2 studies. North America had insufficient data to determine the prevalence of SCD. I^2^ describes the percentage of variation across studies that was due to heterogeneity rather than chance, scored from 0 to 100%, in which 100% is maximum heterogeneity. BN, binomial normal; CI, confidence interval; SCD, sickle cell disease.

**Figure 3:**
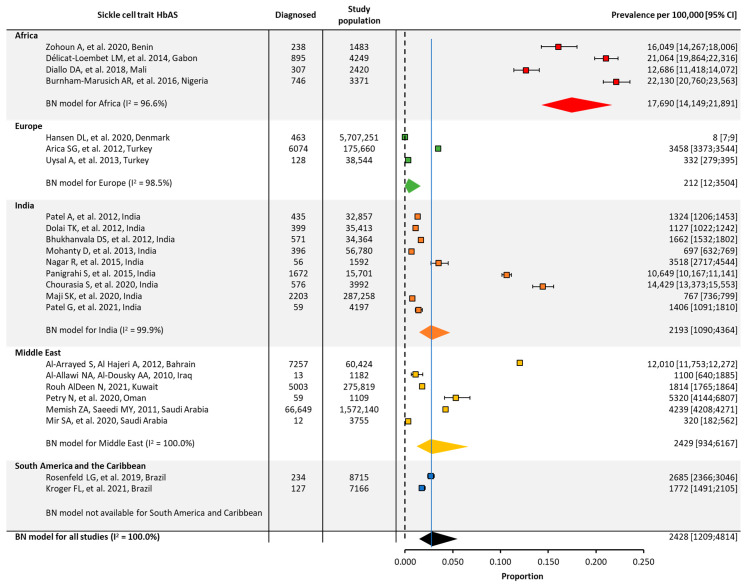
Global and regional prevalence ^a^ of sickle cell trait in Africa [22,23,24,41], Europe [26,28,42], India [30,31,32,33,34,35,43,44,45], the Middle East [36,37,38,39,46,47], and South America/the Caribbean [48,49]. ^a^ Within each region, the prevalence was estimated using a binomial normal model, which assumed a binomial distribution for the individual studies with a mean value drawn from a normal distribution for a regional/global value. The prevalence for each reference was determined from the log odds. A summary estimate was determined for each region with >2 studies. North America had insufficient data to determine the prevalence of SCD. I^2^ describes the percentage of variation across studies that was due to heterogeneity rather than chance, scored from 0 to 100%, in which 100% is maximum heterogeneity. BN, binomial normal; CI, confidence interval; SCD, sickle cell disease.

**Figure 4:**
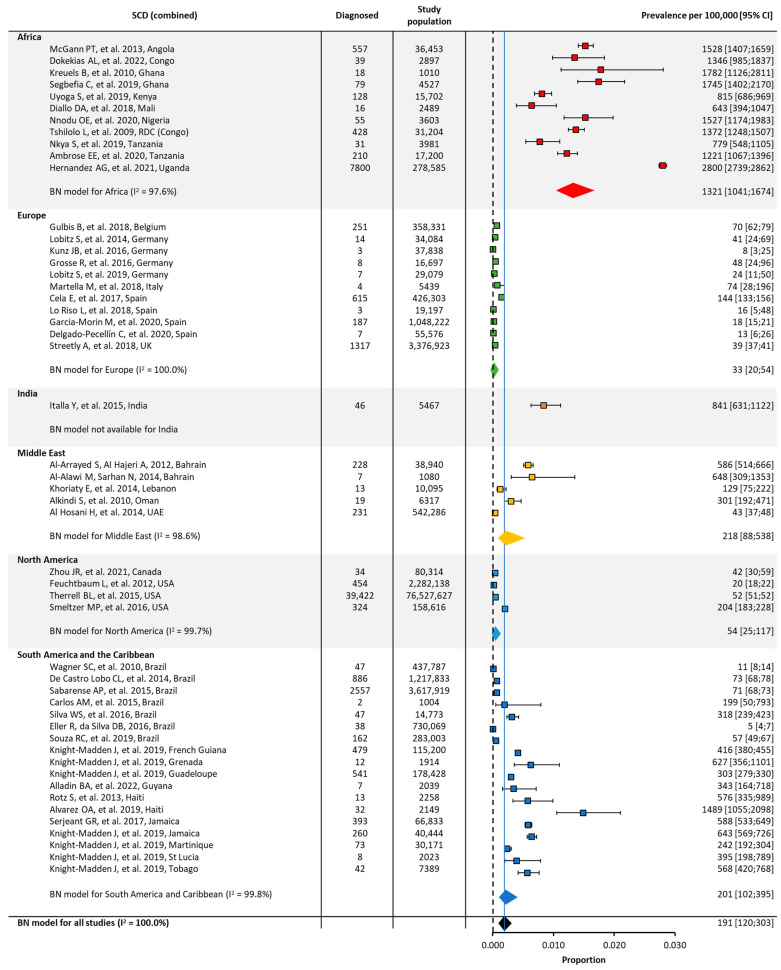
Global and regional birth prevalence ^a^ of SCD in Africa [23,50,51,52,53,54,55,56,57,58,59], Europe [60,61,62,63,64,65,66,67,68,69,70], India [71], the Middle East [39,72,73,74,75], North America [76,77,78,79], and South America/the Caribbean [80,81,82,83,84,85,86,87,88,89,90,91]. ^a^ Within each region, the birth prevalence was estimated using a binomial normal model, which assumed a binomial distribution for the individual studies with a mean value drawn from a normal distribution for a regional/global value. The prevalence for each reference was determined from the log odds. A summary estimate was determined for each region with >2 studies. I^2^ describes the percentage of variation across studies that was due to heterogeneity rather than chance, scored from 0 to 100%, in which 100% is maximum heterogeneity. BN, binomial normal; CI, confidence interval; SCD, sickle cell disease.

**Figure 5:**
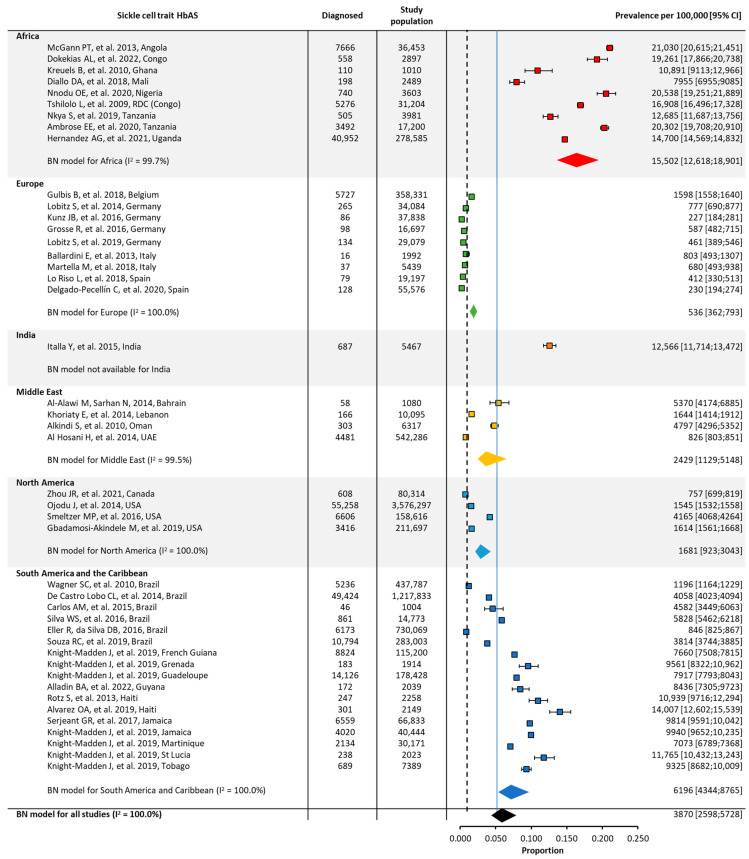
Global and regional birth prevalence ^a^ of sickle cell trait in Africa [23,50,51,52,53,54,57,58,59], Europe [61,63,65,66,67,68,69,70,92], India [71], the Middle East [72,73,74,75], North America [76,79,93,94], and South America/the Caribbean [80,81,82,83,85,86,87,88,89,90,91]. ^a^ Within each region, the birth prevalence was estimated using a binomial normal model, which assumed a binomial distribution for the individual studies with a mean value drawn from a normal distribution for a regional/global value. The prevalence for each reference was determined from the log odds. A summary estimate was determined for each region with >2 studies. I^2^ describes the percentage of variation across studies that was due to heterogeneity rather than chance, scored from 0 to 100%, in which 100% is maximum heterogeneity. BN, binomial normal; CI, confidence interval; SCD, sickle cell disease.

Quantitative analyses on the global and regional prevalence and birth prevalence of SCD and SCT are shown in Figure 2, Figure 3, Figure 4 and Figure 5.

SCD prevalence was highest in Africa (~800/100,000), followed by the Middle East (~200/100,000) and India (~100/100,000). Prevalence was lower in Europe (30/100,000) and could not be calculated for North America or South America/the Caribbean due to a low number of eligible studies (Table 1, Figure 2).

SCT prevalence followed a similar pattern to that of SCD, with the highest prevalence in Africa (~18,000/100,000), followed by the Middle East (~2000/100,000), India (~2000/100,000), and Europe (~200/100,000). The prevalence of SCT could not be calculated for North America or South America and the Caribbean due to the insufficient number of papers from these regions (Table 1, Figure 3).

The birth prevalence of SCD was highest in Africa (~1000/100,000) and lowest in North America (~50/100,000) and Europe (~30/100,000) (Table 1, Figure 4). Similarly, the birth prevalence of SCT was highest in Africa (~16,000/100,000) and lowest in North America (~2000/100,000) and Europe (~500/100,000) (Table 1, Figure 5). The birth prevalence for SCD and SCT for India were not calculated, as only one study included these data. The birth prevalence of SCD reported in this study was ~800/100,000, and the birth prevalence of SCT was ~13,000/100,000 (Table 1, Figure 4 and Figure 5).

As expected, there was considerable heterogeneity (I^2^) in the overall prevalence or birth prevalence for a given region, due to the low number of identified eligible studies; a wide variation across studies, even within the same country; and differing methods of data collection (e.g., the screening of subpopulations or in specific settings such as military drafts or school students versus database studies) (Figure 3, Figure 4 and Figure 5).

**Figure 6:**
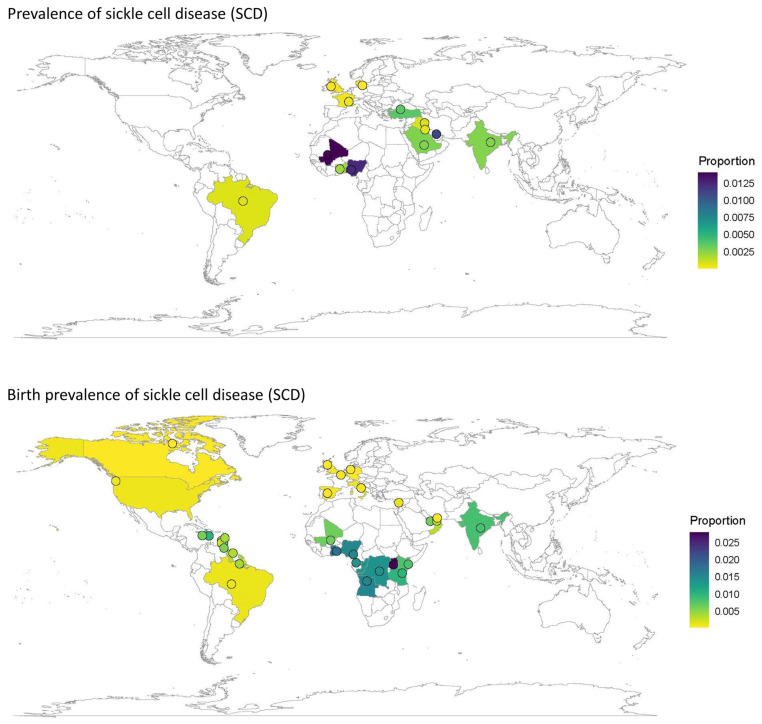
Worldwide prevalence ^a^ and birth prevalence of SCD. ^a^ Each country with results for the specific genotype and prevalence measure are colored according to the reported prevalence (or average if multiple results were available for the given country). Dots are placed at the center of each country (and therefore do not indicate a specific location within the country) and may have been marginally moved in a random direction in order to be able to see multiple dots if the countries are close together. SCD, sickle cell disease.

**Figure 7:**
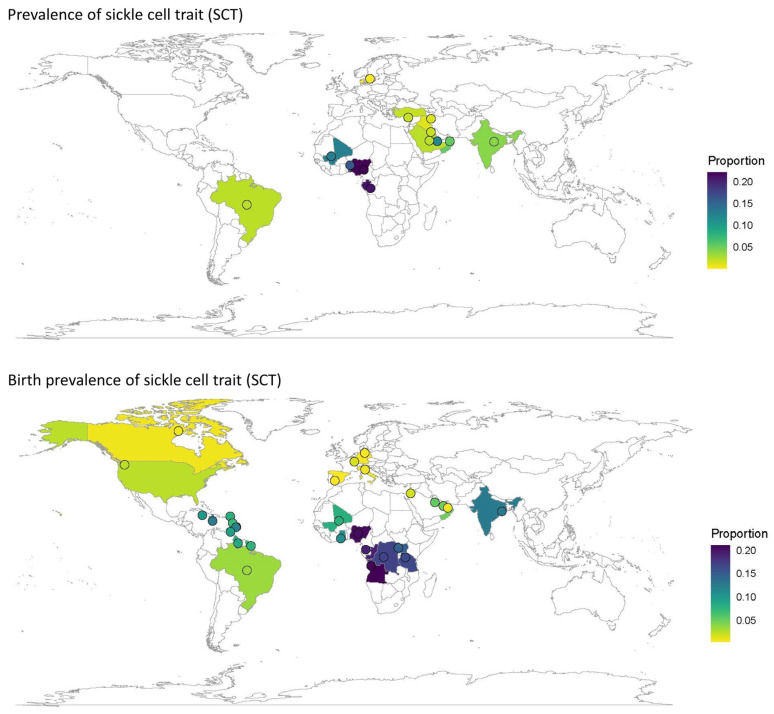
Worldwide prevalence and birth prevalence of sickle cell trait. Each country with results for the specific genotype and prevalence measure are colored according to the reported prevalence (or average if multiple results were available for the given country). Dots are placed at the center of each country (and therefore do not indicate a specific location within the country) and may have been marginally moved in a random direction in order to be able to see multiple dots if the countries are close together.

The prevalence and birth prevalence of SCD (Figure 6) and SCT (Figure 7) plotted by country on a world map visually represents the regions in which the data gaps on any type of prevalence exist. There were no large-scale published data on SCD prevalence from North America; however, some birth prevalence data were available, including one large study of case findings from newborn screening across the US. Large-scale studies of prevalence were available for a few countries in Northern Europe, but only smaller studies from key countries in Southern Europe with an expected increasing prevalence due to migration, such as France, Italy, and Spain (Figure 6).

SCD prevalence data from Africa were also sparse (three countries), while SCD birth prevalence was available from eight African countries (Figure 6). SCD prevalence data for South America and the Caribbean were only available from a single study in Brazil. Although SCD birth prevalence data were available from seven studies in Brazil and one study including a number of Caribbean countries, the majority of the data came from Brazilian publications (Figure 4). SCD prevalence was reported for nine studies in India, but only one study reported SCD birth prevalence (Figure 3).

Prevalence and birth prevalence by genotype (HbSS, HbSC, and sickle β-thalassemia), where these data were available, are reported in Figures S1–S6.

### 3.3. Mortality

**Figure 8:**
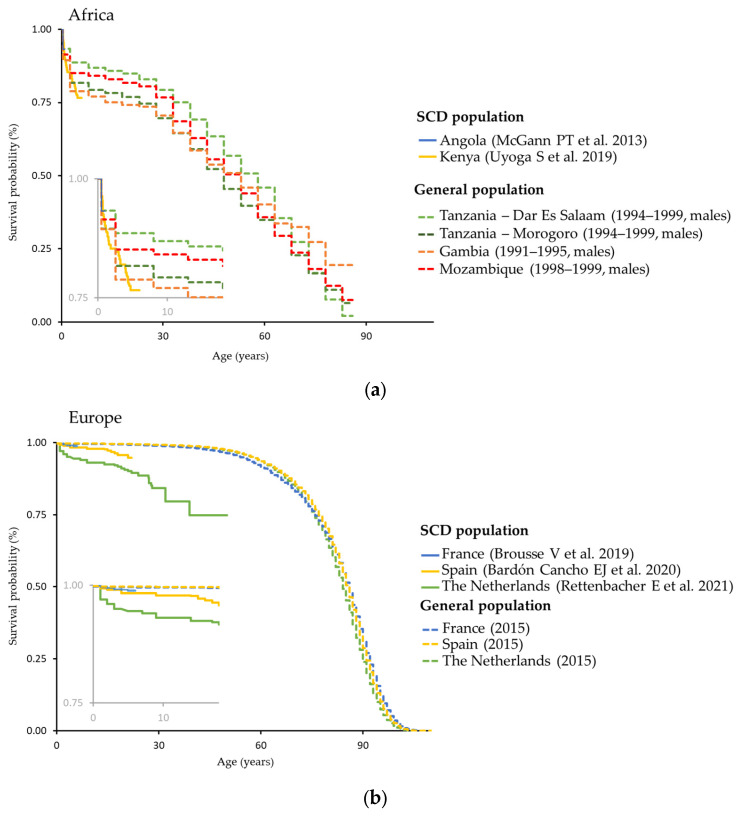

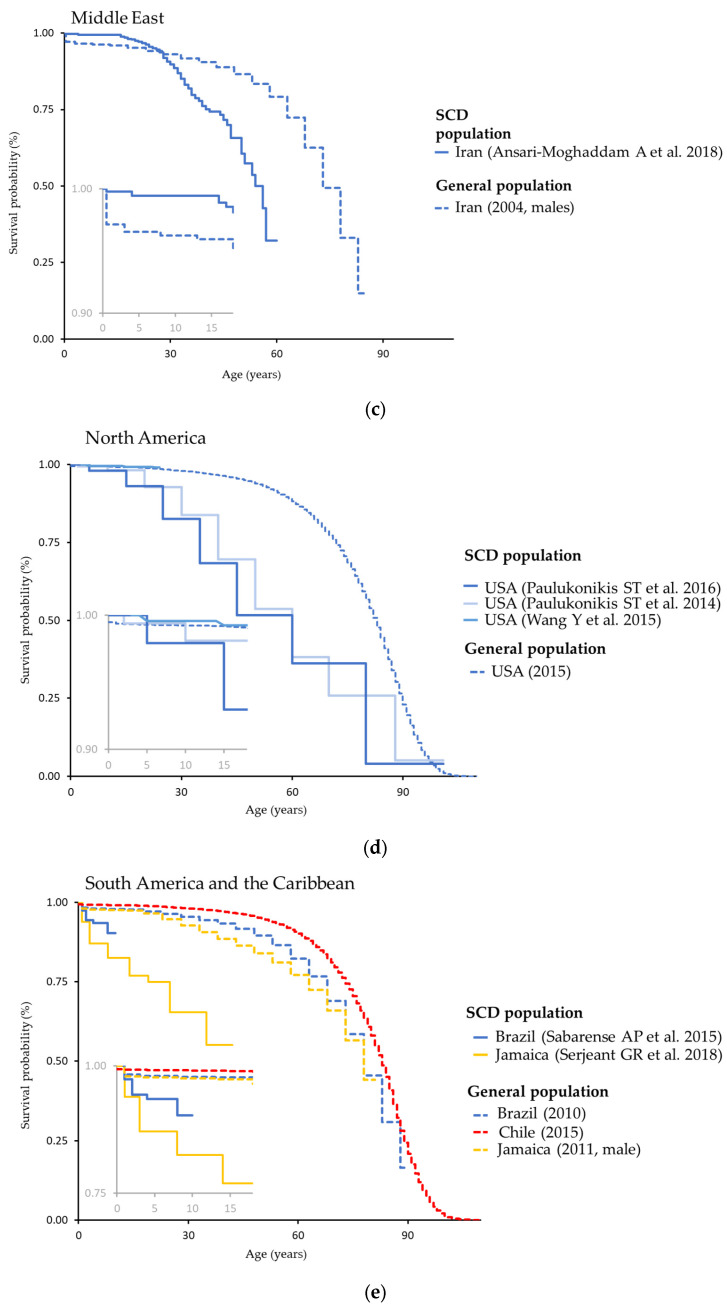
Survival curves (^a^,^b^) for all ages and for the first 15 years of life (inset) for people with SCD and matched general populations in: (**a**) Africa, (**b**) Europe, (**c**) the Middle East, (**d**) North America, and (**e**) South America and the Caribbean. (**a**) Mortality curves for SCD populations from Angola 2013 [59]; Kenya 2019 [55]; and for the general population: Tanzania—Dar Es Salaam 1994–1999 [21,95], Tanzania—Morogoro 1994–1999 [21,95], Gambia 1991–1995 [21,95], and Mozambique. (**b**) Mortality curves for SCD populations from France 2019 [96]; Spain 2020 [97]; the Netherlands 2021 [98]; and for the general population: France 2015 [20], Spain 2015 [20], and the Netherlands 2015 [20]. (**c**) Mortality curves for SCD populations from Iran 2018 [99] and for the general population: Iran 2004 [100]. (**d**) Mortality curves for SCD populations from USA 2014 [101]; USA 2015 [102]; USA 2016 [103]; and for the general population: USA 2015 [20]. (**e**) Mortality curves for SCD populations from Brazil 2015; Jamaica 2018; and for the general population: Brazil 2010 [21,104], Jamaica 2011 [21,105], and Chile 2015 [20]. (^a^) Steps occur at each death (if available) or end of age intervals (for age-binned data). (^b^) Mortality data for the general population were obtained from matched sources for the same country as and similar year(s) to the SCD study. If datasets that matched geographical locations and years were unavailable, data from neighboring countries covering the same geographical area or close in time to the SCD study were used. SCD, sickle cell disease.

Survival curves were determined for Africa, Europe, the Middle East, North America, and South America and the Caribbean, as presented in Figure 8. Each plot presents survival curves for SCD populations, from the studies referenced, and from the general population for comparison [19,20] for the included countries. Generally, observed survival in the SCD population was lower than for the general population. The difference between the SCD population and overall population tended to be larger for regions with less access to healthcare (e.g., Africa) compared with those that had higher access (e.g., North America or Europe).

In the nine studies where the cause of death was reported, the leading causes of death were aligned with the known complications of SCD and included acute chest syndromes (61/345 [17.7%]), various infections (40/345 [11.6%]), sepsis or septicemia (34/345 [9.9%]), splenic sequestration (28/345 [8.1%]), and stroke (26/345 [7.5%]) (Table S8).

## 4. Discussion

As expected, SCD prevalence and/or birth prevalence were highest in Africa, followed by the Middle East, India, and the Caribbean. The prevalence data for South America and the Caribbean were limited by the low number of eligible studies in the region, although the existing studies covered a range of Caribbean countries. For SCT, these relationships were preserved for Africa and India, but not for the Middle East. There could be an actual difference in the SCT to SCD ratio across regions due, for example, to differing mortality rates from SCD. However, this apparent difference may also be due to missing data in high-prevalence areas or may reflect different data gathering methods used in the publications; for example, in the Middle East region, a high portion of publications were of premarital screening studies, which were not widely seen in other regions. Having multiple data sources from individual countries of interest within a specific region (i.e., those with significant expected effects on healthcare resources, such as countries in Europe with an expected increase in prevalence due to migration), would be highly beneficial to allow the calculation of an accurate prevalence and absolute number of people living with SCD in any given country. Although both our study and other previous SLRs showed certain trends in terms of key countries and hotspot regions, differences between countries and regions observed here were likely not wholly driven by geography. There are other possible contributing factors such as differences in study protocols and population size. With multiple potential sources of bias affecting the accuracy of the prevalence estimates for each country, reliable absolute numbers could not be calculated for countries of interest with the data currently available.

A previous SLR by Wastnedge et al. [12], which included 67 papers from January 1980 to July 2017 in a meta-analysis, gave a global estimate for the birth prevalence of homozygous SCD of 112/100,000 live births (95% CI 101–123). In Africa, the birth prevalence was 1125/100,000 (95% CI 680–1570) compared with 43/100,000 (95% CI 30–56) in Europe [12]. While the findings of the current study were broadly in line with the results of this meta-analysis, with Africa as an SCD hotspot, the Wastnedge et al. review included prevalence data that covered over three decades [12]. This SLR included publications from 2010 onwards, as it was considered that epidemiological and mortality data from older publications would not be representative of the current situation, considering changes over time in healthcare practices and the prevalence and disease burden of SCD in various regions arising from global population movements [2].

A study by Piel et al. [10] using a database of sickle hemoglobin surveys to estimate and map the global distribution of neonates with SCT (HbAS) or homozygous SCD (HbSS) showed that high frequencies (>10%) of the HbS allele (i.e., HbAS plus HbSS) were almost exclusively encountered in sub-Saharan Africa, with additional relatively high frequencies in India [10]. However, Piel et al. covered only neonate estimates, focusing on HbAS and HbSS, and did not include SCD birth prevalence from other SCD genotypes, meaning their study did not cover the estimation of healthcare needs for all aspects of the disease. While the previous SLRs focused on birth prevalence, this current SLR addressed some of the limitations of these earlier SLRs by investigating both prevalence and updated estimates for birth prevalence. The current SLR also included a wider range of both SCD genotypes and SCT in the search terms; however, data on the rare or regional forms were limited. The individual genotypes were described in the synthesized qualitative overview but were included in the overall SCD groups for the meta-analyses. Furthermore, most publications reporting on the prevalence and birth prevalence of HbS/β-thalassemia unfortunately did not separate the more severe form of HbS/β0-thalassemia and the less severe form of HbS/β+-thalassemia, a granularity that would be important for an accurate understanding of the healthcare needs related to SCD in a specific region.

The current SLR revealed important gaps in the published literature, with missing data on prevalence and/or birth prevalence from countries where SCD is believed to be highly prevalent, including African countries such as Cameroon and Mozambique. In addition, although some identified studies represented large African countries, such as Nigeria, Egypt, and the Democratic Republic of Congo, the data were limited due to the total study populations being in the 10,000s. There were also limited or no data from other regions where prevalence is expected to be on the rise through migration and higher birth rates of the migrant population relative to the native population, such as Europe and North America. Up-to-date information from these regions is important to understand the changing prevalence in these areas in order to ensure early detection, adequate prevention programs, and appropriate healthcare resources.

Countries with a very low prevalence, for example, countries in Eastern Europe, would likely not collect data on SCD, as the disease does not pose a significant clinical issue in these countries. However, in some countries with a low or very heterogeneous prevalence of SCD in the population, screening programs are often limited to at-risk populations, for example, members of specific ethnic groups. For instance, Mueller et al. [106] reported on SCD prevalence in Ghanaian migrants in Germany, where the prevalence of SCT and SCD were 17.4% and 1.2%, respectively, much higher than the estimates reported for the general German population [107]. With the rising prevalence in Germany, a nationwide registry of patients across centers caring for children with SCD has been established to understand specifically the disease burden and patient needs in Germany [108]. Similarly, in India, screening for sickle cell disease is carried out among children in tribal schools to allow the development of genetic counselling and intervention programs to reduce morbidity and mortality [30,35,109,110]. Although these studies may not be representative of the general population and are often not large enough to be included in meta-analyses, in the absence of the screening of the whole population they may provide important insights into the prevalence of SCD among certain subpopulations in the respective regions.

Mortality, life-expectancy, and survival data were limited, as publications reporting these were sparse, and the information was reported in many different ways (e.g., by median length of life, mean length of life, life expectancy, and survival time). The causes of death reported by the identified publications generally aligned with key complications of the SCD pathology, such as thrombosis/sickle cell crises and ensuing tissue/organ damage, as well as an increased susceptibility to infections [3]. As noted in other aspects of data collection in the field of SCD, gaps in the data on the cause of death were prevalent, with ~25% (8/345) of deaths across the nine publications being reported as other/not known.

Furthermore, SCD genotypes, diagnostic criteria, and study settings differed widely between publications that included mortality data. However, for a number of studies, it was possible to present mortality data as survival curves. In general, survival observed in the SCD population was lower than what was reported for the general population. Furthermore, a trend could be observed towards a larger difference for regions with less access to healthcare. This difference was particularly noticeable in Africa (Angola and Kenya), where mortality in infancy was elevated in the first 5 years of life. However, no data were available for older children or adults. Unfortunately, the reasons for differences in the cause of death between people with SCD and the general population were hard to ascertain, as only nine of the 115 identified publications reported causes of death, and only in a few of these cases did the same paper report on mortality and causes of death. The complications of SCD tend to differ in children and adults; the available data did not allow for the further confirmation of causes of death across age groups.

A limitation of a meta-analysis based on the data in this SLR would be that there were few studies in some regions, such as North America. A number of US studies identified in the search were excluded from the analyses as they were from a single center only, which would make them difficult to generalize to the broader population of the country. In addition, there was considerable heterogeneity between countries within regions and even within countries, for example in Brazil and India [111]. Given the large inhomogeneity (I^2^)—close to 100%—the regional/global results were more difficult to interpret and should be used with caution. The main reason for the large inhomogeneity was the actual differences between populations across regions and/or different groups across countries. In addition, the inhomogeneity could have also been due to differences in sampling methods, imprecise estimations in individual studies, or different subpopulations.

The limitations to this SLR were mainly due to the identified gaps in the published literature, including the inconsistent reporting of the different SCD genotypes, variability in diagnostic criteria, and different study settings. There was a clear deficit of studies from some large regions of the world (e.g., US, Russia, Asian countries apart from India) in the current SLR. The scarce information published over the 12-year study period highlights the need to scale up systematic epidemiological data collection on SCD and SCT by registries, databases, and longitudinal studies using specific and clearly defined parameters to properly estimate the disease burden.

Few countries reported both prevalence and birth prevalence. The studies on birth prevalence (i.e., prevalence in children <1 year of age) were mainly based on newborn screening programs. Newborn screening data have advantages like standardized data collection and are high-quality, providing a good basis for estimating future care needs. However, for a better estimate of care needs in any given population, we would need to combine the birth prevalence data with up-to-date survival data in the SCD population from the equivalent region/country. With the great heterogeneity in the research on mortality identified in the current SLR, it is clear that further research would be needed to determine up-to-date and adequate prevention and screening strategies, targeting the causes of SCD mortality. The Rare Anaemia Disorders European Epidemiological Platform (RADeep, https://www.radeepnetwork.eu/, accessed on 21 August 2023) aims to set up a European registry of patients affected by rare anemia diseases by creating a network of centers and registries and establishing a framework for sharing data. Similarly, the Sickle Pan-African Research Consortium (SPARCO) has started enrolling patients into a centralized hemoglobinopathy database for the collection of longitudinal data [112]. We hope that these initiatives will increase the amount of comprehensive data collection for all genotypes, which this SLR demonstrated to be lacking at the moment. This could be achieved by strengthening uniform and standardized routine data collection.

## 5. Conclusions

This SLR confirmed earlier studies of areas with a high prevalence of SCD but identified important data gaps. Resources are needed for additional studies across regions to provide uniform data collection on prevalence and mortality, ensuring increased SCD awareness among healthcare professionals and public health policymakers worldwide.

## Figures and Tables

**Table 1:** Quantitative analysis of SCD and SCT for prevalence and birth prevalence.

**Prevalence**								
**SCD**					**SCT**			
**Region**	**No. of Studies**	**Total Studied Population**	**Prevalence** **per 100,000** **[95% CI]**			**No. of Studies**	**Total Studied Population**	**Prevalence** **per 100,000** **[95% CI]**
Global	19	130,420,748	117 [55;249]			24	8,335,442	2428 [1209;4814]
Africa	3	7274	788 [316;1951]			4	11,523	17,690 [14,149;21,891]
Europe	5	127,536,172	28 [10;79]			3	5,921,455	212 [12;3503]
India	6	430,952	128 [48;340]			9	472,154	2193 [1090;4364]
Middle East	4	1,909,565	212 [64;698]			6	1,914,429	2429 [934;6167]
North America	–	–	NA			–	–	NA
South America/ the Caribbean	1	536,785	NA			2	15,881	NA
**Birth Prevalence**								
**SCD**				**SCT**				
**Region**	**No. of Studies**	**Total Studied Population**	**Prevalence ** **per 100,000** **[95% CI]**			**No. of Studies**	**Total Studied Population**	**Prevalence ** **per 100,000** **[95% CI]**
Global	44	92,209,456	191 [120;303]			44	8,661,141	3870 [2598;5728]
Africa	11	397,651	1321 [1041;1674]			9	377,422	15,502 [12,618;18,901]
Europe	11	5,407,689	33 [20;54]			9	558,233	535 [362;792]
India	1	5467	NA			1	5467	NA
Middle East	5	598,718	218 [88;538]			4	559,778	2429 [1129;5148]
North America	4	79,048,695	54 [25;117]			4	4,026,924	1681 [923;3043]
South America/ the Caribbean	12	6,751,236	201 [102;395]			11	3,133,317	6196 [4344;8765]

Within each region, the prevalence was estimated using a binomial normal model, which assumed a binomial distribution for the individual studies with a mean value drawn from a normal distribution for a regional/global value. A summary estimate was determined for each region with >2 studies. CI, confidence interval; NA, not available; SCD, sickle cell disease; SCT, sickle cell trait.

## Data Availability

Data may be available on reasonable request from the authors.

## Supplementary Materials

The following supporting information can be downloaded at: https://www.mdpi.com/2077-0383/12/17/5538/s1, Table S1: Search terms and strategy for bibliographic databases, Table S2: Study eligibility criteria according to PICOS, Table S3: Categories of data extraction captured on data extraction dashboard based on Excel, Table S4: Synthesis of regional prevalence of overall SCD and specific genotypes, Table S5: Synthesis of regional prevalence of SCT (HbAS), Table S6: Synthesis of regional birth prevalence of overall SCD and specific genotypes, Table S7: Synthesis of regional birth prevalence of SCT (HbAS), Table S8: Causes of death among people with sickle cell disease, Figure S1: Global and regional prevalence of homozygous SCD (HbSS), Figure S2: Global and regional prevalence of heterozygous SCD (HbSC), Figure S3: Global and regional prevalence of sickle cell β-thalassemia, Figure S4: Global and regional birth prevalence of homozygous SCD (HbSS), Figure S5: Global and regional birth prevalence of heterozygous SCD (HbSC), Figure S6: Global and regional birth prevalence of sickle cell β-thalassemia. References [113,114,115,116,117,118,119,120,121,122,123,124,125,126,127,128,129,130,131,132,133,134,135,136,137,138,139,140,141,142,143,144,145,146,147,148,149,150,151,152,153,154].
