# Correction: Colombatti et al. Systematic Literature Review Shows Gaps in Data on Global Prevalence and Birth Prevalence of Sickle Cell Disease and Sickle Cell Trait: Call for Action to Scale Up and Harmonize Data Collection. *J. Clin. Med*. 2023, *12*, 5538

**DOI:** 10.3390/jcm13102893

**Published:** 2024-05-14

**Authors:** Raffaella Colombatti, Inga Hegemann, Morten Medici, Camilla Birkegård

**Affiliations:** 1 Clinic of Pediatric Hematology Oncology, Department of Child and Maternal Health, Azienda Ospedaliera, University of Padova, 35122 Padua, Italy; 2 Novo Nordisk Pharma AG, 8058 Zürich, Switzerland; inhm@novonordisk.com; 3 Novo Nordisk A/S, 2860 Søborg, Denmark; vmei@novonordisk.com (M.M.); acbk@novonordisk.com (C.B.)

## Error in Figure/Table

In the original publication [1], there was a mistake in Table 1. The total number of studies and the total study population for the prevalence of SCD in Europe, the global birth prevalence and the global prevalence of SCT per 100,000 were amended. The corrected Table 1 appears below.

In the original publication, there was a mistake in Figure 2. The heterogeneity (I^2^) number for Europe and the confidence intervals have been adjusted. A reference citation for the Middle East has also been updated. The corrected Figure 2 appears below.

In the original publication, there was a mistake in Figure 3. The confidence intervals, the heterogeneity (I^2^) number for Europe, and the overall prevalence number have been adjusted. Additionally, a reference citation for the Middle East has been amended. The corrected Figure 3 appears below.

In the original publication, there was a mistake in Figure 4. The confidence intervals, one prevalence number for Africa, the heterogeneity (I^2^) number for Europe, and the overall I^2^ number have been adjusted. A reference citation for the Middle East has also been updated. The corrected Figure 4 appears below.

In the original publication, there was a mistake in Figure 5. One prevalence number for Europe, the confidence intervals, and one heterogeneity (I^2^) number for Europe have been adjusted. The corrected Figure 5 appears below.

The authors state that the scientific conclusions are unaffected. These corrections were approved by the Academic Editor. The original publication has also been updated.

## Figures and Tables

**Figure 2:**
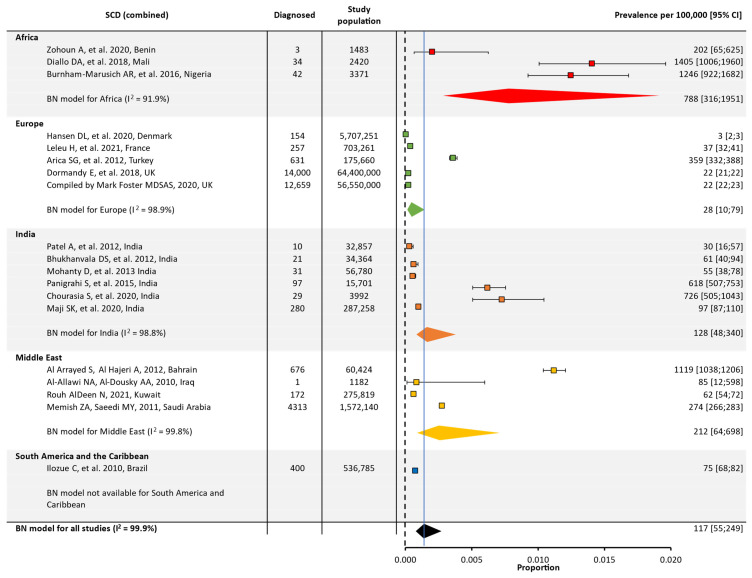
Global and regional prevalence ^a^ of SCD in Africa [22–24], Europe [25–29], India [30–35], the Middle East [36–39], and South America/the Caribbean [40]. ^a^ Within each region, the prevalence was estimated using a binomial normal model, which assumed a binomial distribution for the individual studies with a mean value drawn from a normal distribution for a regional/global value. The prevalence for each reference was determined from the log odds. A summary estimate was determined for each region with >2 studies. North America had insufficient data to determine the prevalence of SCD. I^2^ describes the percentage of variation across studies that was due to heterogeneity rather than chance, scored from 0 to 100%, in which 100% is maximum heterogeneity. BN, binomial normal; CI, confidence interval; SCD, sickle cell disease.

**Figure 3:**
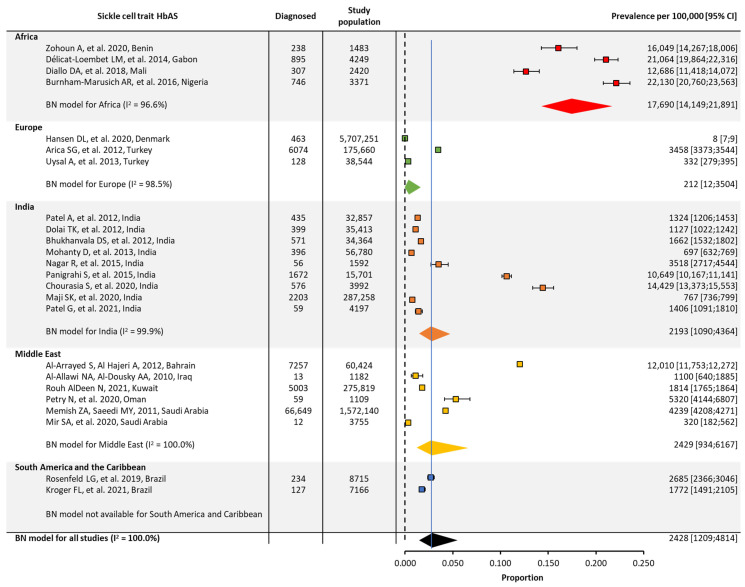
Global and regional prevalence ^a^ of sickle cell trait in Africa [22–24,41], Europe [26,28,42], India [30–35,43–45], the Middle East [36–39,46,47], and South America/the Caribbean [48,49]. ^a^ Within each region, the prevalence was estimated using a binomial normal model, which assumed a binomial distribution for the individual studies with a mean value drawn from a normal distribution for a regional/global value. The prevalence for each reference was determined from the log odds. A summary estimate was determined for each region with >2 studies. North America had insufficient data to determine the prevalence of SCD. I^2^ describes the percentage of variation across studies that was due to heterogeneity rather than chance, scored from 0 to 100%, in which 100% is maximum heterogeneity. BN, binomial normal; CI, confidence interval; SCD, sickle cell disease.

**Figure 4:**
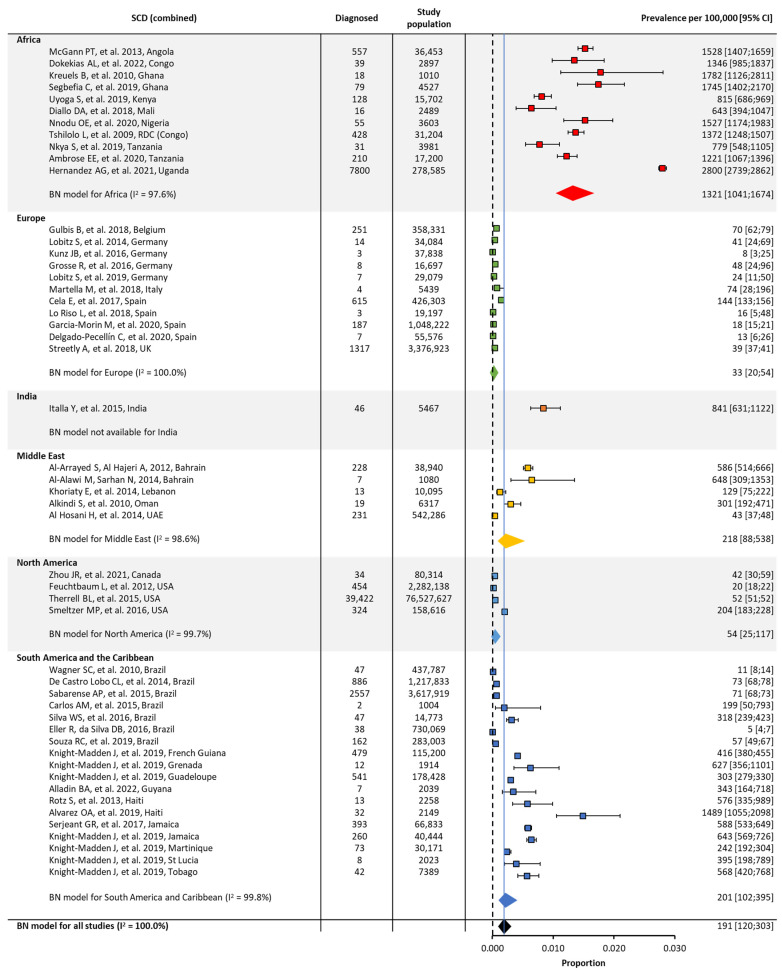
Global and regional birth prevalence ^a^ of SCD in Africa [23,50–59], Europe [60–70], India [71], the Middle East [39,72–75], North America [76–79], and South America/the Caribbean [80–91]. ^a^ Within each region, the birth prevalence was estimated using a binomial normal model, which assumed a binomial distribution for the individual studies with a mean value drawn from a normal distribution for a regional/global value. The prevalence for each reference was determined from the log odds. A summary estimate was determined for each region with >2 studies. I^2^ describes the percentage of variation across studies that was due to heterogeneity rather than chance, scored from 0 to 100%, in which 100% is maximum heterogeneity. BN, binomial normal; CI, confidence interval; SCD, sickle cell disease.

**Figure 5:**
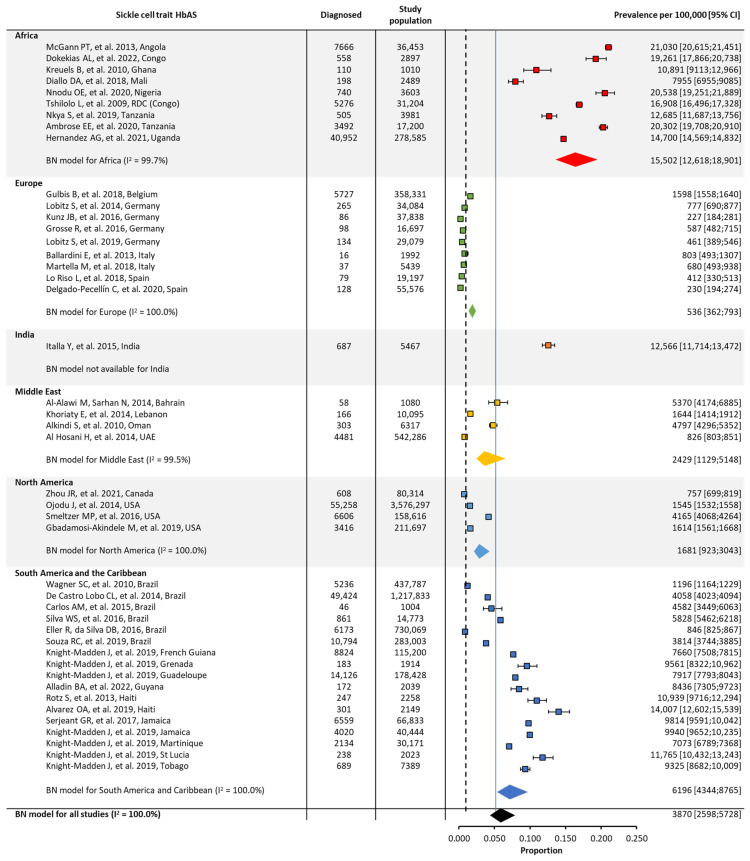
Global and regional birth prevalence ^a^ of sickle cell trait in Africa [23,50–54,57–59], Europe [61,63,65–70,92], India [71], the Middle East [72–75], North America [76,79,93,94], and South America/the Caribbean [80–83,85–91]. ^a^ Within each region, the birth prevalence was estimated using a binomial normal model, which assumed a binomial distribution for the individual studies with a mean value drawn from a normal distribution for a regional/global value. The prevalence for each reference was determined from the log odds. A summary estimate was determined for each region with >2 studies. I^2^ describes the percentage of variation across studies that was due to heterogeneity rather than chance, scored from 0 to 100%, in which 100% is maximum heterogeneity. BN, binomial normal; CI, confidence interval; SCD, sickle cell disease.

**Table 1:** Quantitative analysis of SCD and SCT for prevalence and birth prevalence.

Prevalence						
	SCD			SCT		
Region	No. of Studies	Total Studied Population	Prevalence per 100,000 [95% CI]	No. of Studies	Total Studied Population	Prevalence per 100,000 [95% CI]
Global	19	130,420,748	117 [55; 249]	24	8,335,442	2428 [1209; 4814]
Africa	3	7274	788 [316; 1951]	4	11,523	17,690 [14,149; 21,891]
Europe	5	127,536,172	28 [10; 79]	3	5,921,455	212 [12; 3503]
India	6	430,952	128 [48; 340]	9	472,154	2193 [1090; 4364]
Middle East	4	1,909,565	212 [64; 698]	6	1,914,429	2429 [934; 6167]
North America	–	–	NA	–	–	NA
South America/ the Caribbean	1	536,785	NA	2	15,881	NA
**Birth Prevalence**						
	**SCD**			**SCT**		
**Region**	**No. of Studies**	**Total Studied Population**	**Prevalence** **per 100,000** **[95% CI]**	**No. of Studies**	**Total Studied Population**	**Prevalence** **per 100,000** **[95% CI]**
Global	44	92,209,456	191 [120; 303]	44	8,661,141	3870 [2598; 5728]
Africa	11	397,651	1321 [1041; 1674]	9	377,422	15,502 [12,618; 18,901]
Europe	11	5,407,689	33 [20; 54]	9	558,233	535 [362; 792]
India	1	5467	NA	1	5467	NA
Middle East	5	598,718	218 [88; 538]	4	559,778	2429 [1129; 5148]
North America	4	79,048,695	54 [25; 117]	4	4,026,924	1681 [923; 3043]
South America/ the Caribbean	12	6,751,236	201 [102; 395]	11	3,133,317	6196 [4344; 8765]

Within each region, the prevalence was estimated using a binomial normal model, which assumed a binomial distribution for the individual studies with a mean value drawn from a normal distribution for a regional/global value. A summary estimate was determined for each region with >2 studies. CI, confidence interval; NA, not available; SCD, sickle cell disease; SCT, sickle cell trait.
