# Supplementary material for: Systematic Literature Review Shows Gaps in Data on Global Prevalence and Birth Prevalence of Sickle Cell Disease and Sickle Cell Trait: Call for Action to Scale Up and Harmonize Data Collection
Source: J Clin Med. 2023 Aug 25;12(17):5538. doi: 10.3390/jcm12175538 (PMC10488271; doi:10.3390/jcm12175538)
Supplement: Supplementary file 1 [file jcm-12-05538-s001.zip › jcm-12-05538-s001.pdf]

Journal of
Clinical Medicine

MDPI

SUPPLEMENTARY MATERIALS

Table S1. Search terms and strategy for bibliographic databases.

Set#	Searched for	Results
S1	MJMESH.EXACT("Anemia, Sickle Cell") OR MJMESH.EXACT("Hemoglobin SC Disease") OR MJMESH.EXACT("Sickle Cell Trait, sickle cell anemia, sickle cell beta thalassemia, sickle cell trait, hemoglobin SC disease")	51,841*
S2	MESH.EXACT("Epidemiology") OR EMB.EXACT("epidemiology") OR EMB.EXACT("incidence") OR MESH.EXACT("Incidence") OR MESH.EXACT("Prevalence") OR EMB.EXACT("prevalence") OR EMB.EXACT("lifetime prevalence") OR EMB.EXACT("newborn mortality") OR EMB.EXACT("mortality") OR EMB.EXACT("infant mortality") OR EMB.EXACT("mortality rate") OR EMB.EXACT("mortality risk") OR MESH.EXACT("Mortality, Premature") OR MESH.EXACT("Child Mortality") OR MESH.EXACT("Mortality") OR MESH.EXACT("Perinatal Mortality") OR MESH.EXACT("Infant Mortality") OR MESH.EXACT("Prognosis")	6,200,951*
S3	MESH.EXACT("Anemia, Sickle Cell -- epidemiology")	2,063*
S4	(S1 AND S2) OR S3	9,757*
S5	Ti,ab("Sickle anaemia" OR "Sickle anemia" OR "Sickle Cell Anaemia" OR "Sickle cell anemia" OR "Sickle cell disease*" OR "Sickle-cell" OR "sickle-anaemia" OR "sickle-anemia" OR "Sickle cell trait" OR "Sickle patient*" OR "sickle C disease" OR "sickle-C disease" OR "sickling" OR "sickled" OR "haemoglobin S beta thalassaemia" OR "haemoglobin S beta thalassemia" OR "hemoglobin S beta thalassaemia" OR "hemoglobin S beta thalassemia" OR "Hb S beta-thalassemia" OR "Hb S beta-thalassemia" OR "Sickle beta + thalassaemia" OR "Sickle beta + thalassemia" OR "sickle beta thalassaemia" OR "sickle beta thalassemia" OR "Sickle beta+ thalassaemia" OR "Sickle beta+ thalassemia" OR "sickle cell beta plus thalassaemia" OR "sickle cell beta plus thalassemia" OR "sickle cell beta thalassaemia" OR "sickle cell beta thalassemia" OR "sickle β-Thalassaemia" OR "sickle ß-Thalassemia") n/3 ti,ab(epidemiology or epidemiologic or fatality or fatalities or incidence or incident or "life expectancy" or "lifetime expectancy" or mortality or prevalence or prevalent or prognosis)	4,391*
S6	Ti,ab(("sickle/beta" OR "SCA" OR "SCD" OR "sickle" OR "Sickle Beta" OR "sickle B" OR "sickle β" OR "Sickle C" OR "Sickled" OR "Sickling") n/3 ("Hemoglobinopath*" OR "Haemoglobinopath*" OR "hemolytic" OR "haemolytic" OR "Homozyg*" OR "heterozyg*" OR "thalassaemia" OR "thalassemia" OR "beta thal"))	17,273*

S7	Ti,ab("SCD HbSC" OR "SCD-HBSC" OR "HB C" or "HB D" OR "HBA" OR "HBAS" OR "HB-AS" OR "HBS" or "HB-S" OR "HBSC" OR "HB-SC" OR HBSS OR "HB-SS") n/3 ti,ab(sickl*)	4,917*
S8	S7 OR S6	20,807*
S9	ti,ab(epidemiology or epidemiologic or fatality or fatalities or incidence or incident or "life expectancy" or "lifetime expectancy" or mortality or prevalence or prevalent or prognosis)	11,485,699*
S10	S9 AND S8	5,290*
S11	S10 OR S5 OR S4	15,923*
S12	(S10 OR S5 OR S4) and (pd(2010-2029))	9,612*
S13	(S10 OR S5 OR S4) and (pd(2010-2029)) not (rtype.exact("Conference Abstract" OR "Review" OR "Meeting Abstract" OR "Letter" OR "Case Reports" OR "Literature Review" OR "Editorial" OR "Note" OR "Meeting Poster" OR "Comment" OR "Conference Paper" OR "Erratum" OR "Editorial Material" OR "Conference Review" OR "Congress" OR "News" OR "Meeting Summary" OR "Correction" OR "Published Erratum" OR "Errata" OR "Interview" OR "Lecture" OR "Retracted Publication" OR "Tombstone" OR "Video-Audio Media"))	5,183*
S14	S13 NOT ti("case report*" OR "case series" OR "biomarker*" OR "oxygen" OR "apnoea" OR "apnea" OR "osteo*" OR "asthma" or respiratory or "orofacial*" OR "dental*" OR "renal" OR "endocrine" or "cardiopulmonary" OR "pulmonary" OR "hypogonadism" or "targeted therapy" OR "gene editing" OR acupuncture OR HIV OR HCV OR HBV OR "electrophoresis" OR "adherence" OR "MRI" OR "radiologic" OR "COVID" or "coronavirus" OR "stem cell*" OR "gene therapy" OR mechanism* OR antigen* OR "transplant*" OR "transfusion*" OR "microrna*" OR "diabetes" or "donor" OR "cytotoxicity" OR "animal*" or "fruit" OR "plum" or "rats" OR "mouse" or "mice" OR "muridae" OR "rodent*" OR "dog*" OR "cat" OR "chimp*" OR "monkey*" OR "primate*" OR "baboon*" or cancer or hypertens* or "chest syndrome" or "mental health" or depression or infection or virus or "B19" or pneumococcal or plasmodium or alloimmunisation or alloimmunization or transfusion or cannabis or marijuana or stroke or coronary or penicillin or renal or malaria or ulcer or pain or nocturnal or acute or Cardiac or Cardiovascular or Cerebral or Cerebrovascular or Chronic or Echocardiographic or Electrocardiographic or "Fat embolism syndrome" or "Glucose-6-phosphate" or "Left ventricular" or "VTE" or "Venous thromboembolism" or "vitamin D" or priapism or "Vasoocclusive crisis" or "VOCs")	1,770†
S15	(S10 OR S5 OR S4) and (rtype.exact("Conference Abstract" OR "Meeting Abstract" OR "Conference Paper" OR "Meeting Paper"))	3,149*
S16	(S10 OR S5 OR S4) and (pd(2010-2029) and (rtype.exact("Conference Abstract" OR "Meeting Abstract" OR "Conference Paper" OR "Meeting Paper")))	2,792

S17	(S10 OR S5 OR S4) and (pd(2018-2021) and (rtype.exact("Conference Abstract" OR "Meeting Abstract" OR "Conference Paper" OR "Meeting Paper")))	1,178
S18	(S10 OR S5 OR S4) and (pd(2018-2021) and (rtype.exact("Conference Abstract" OR "Meeting Abstract" OR "Conference Paper" OR "Meeting Paper")))	696
S19	S18 NOT ti("case report*" OR "case series" OR "biomarker*" OR "oxygen" OR "apnoea" OR "apnea" OR "osteo*" OR "asthma" or respiratory or "orofacial*" OR "dental*" OR "renal" OR "endocrine" or "cardiopulmonary" OR "pulmonary" OR "hypogonadism" or "targeted therapy" OR "gene editing" OR acupuncture OR HIV OR HCV OR HBV OR "electrophoresis" OR "adherence" OR "MRI" OR "radiologic" OR "COVID" or "coronavirus" OR "stem cell*" OR "gene therapy" OR mechanism* OR antigen* OR "transplant*" OR "transfusion*" OR "microrna*" OR "diabetes" or "donor" OR "cytotoxicity" OR "animal*" or "fruit" OR "plum" or "rats" OR "mouse" or "mice" OR "muridae" OR "rodent*" OR "dog*" OR "cat" OR "chimp*" OR "monkey*" OR "primate*" OR "baboon*" or cancer or hypertens* or "chest syndrome" or "mental health" or depression or infection or virus or "B19" or pneumococcal or plasmodium or alloimmunisation or alloimmunization or transfusion or cannabis or marijuana or stroke or coronary or penicillin or renal or malaria or ulcer or pain or nocturnal or acute or Cardiac or Cardiovascular or Cerebral or Cerebrovascular or Chronic or Echocardiographic or Electrocardiographic or "Fat embolism syndrome" or "Glucose-6-phosphate" or "Left ventricular" or "VTE" or "Venous thromboembolism" or "vitamin D" or priapism or "Vasoocclusive crisis" or "VOCs")	468[†]

*Duplicates are removed from the search but included in the result count. [†]Duplicates are removed from the search and from the result count. Hb, hemoglobin; HIV, human immunodeficiency virus; MRI, magnetic resonance imaging; SC, sickle cell; SCA, sickle cell anemia; SCD, sickle cell disease; VOC, vaso-occlusive crisis; VTE, venous thromboembolism.

Table S2. Study eligibility criteria according to PICOS.

Criteria	Description
**Population**	• People of any age with any form of SCD
**Interventions**	• Not applicable (epidemiological studies)
**Comparators**	• Not applicable (epidemiological studies)
**Outcomes**	Studies must report at least one of the following outcomes:<br>• Prevalence<br>• Birth prevalence<br>• Life expectancy<br>• Mortality related to SCD and its complications
**Considerations**	• All studies apart from the following exclusions:<br>• Animal studies, *in vitro*/*ex vivo* studies, gene/protein expression studies, or modeling studies<br>• Single-center studies or those reporting data from a specific geographical sub-region within a country

		• Conference or meeting abstracts, meeting posters, or conference papers older than 2018<br>• Conference or meeting abstracts, meeting posters, or conference papers; reviews and literature reviews; letters, case reports, editorials, comments, or editorial material; news, interviews, lectures, video-audio material, tombstone advertisements, or meeting summary; or errata, correction, or retracted publication<br>• Duplicates; for studies published in more than one report, the most comprehensive and up-to-date version will be used
**Time**		• January 1, 2010 and March 25, 2022
**Language**		• Studies published in English, French, and Spanish, or other languages with at least an English abstract will be included<br>• Abstracts of non-English/French/Spanish publications captured in the search will be reviewed to ensure they do not include relevant outcome data from countries with few identified publications

PICOS, Population, Intervention, Comparison, Outcomes and Study; SCD, sickle cell disease.

Table S3. Categories of data extraction captured on data extraction dashboard based on Excel.

Parameter	Details
Authors, year, and title of publication	Free text
Year(s) of data collection	Specify
Greater region/country/region	Africa, Europe, Middle East, North America, India, South America/other (specify), France, Germany/Italy, India, UK, US/specify in free text
Study design	Prospective, Retrospective
Type of study	Cohort, Cross-section, Newborn screening, Registry, Other (free text)
Race/ethnicity	Free text
Number of people with SCD	Free text
Total population at risk	Free text
Disease classification (genotype)	Homozygous (HbSS), Heterozygous (HbSC), Sickle beta thalassemia, SCD (genotype unknown), Sickle beta-zero thalassemia, Sickle cell trait (HbAS)
Proportion male (%)	Free text
Data source	Electronic medical records, Claims database, Payor database, Other (specify in free text)
Population source	Hospital, General population, Other (specify in free text)
Age group	Pediatric (<18 years), Adult (18–64 years), Elderly (≥65 year), General, Not stated
Mean/median age of the study population	Free text
Statistical modeling	Crude/unadjusted rate, Adjusted rate
Prevalence data	Prevalence (cases/total population), Prevalence (%)
Birth prevalence data	Birth prevalence (cases/total newborns), Birth prevalence estimate (%)
Mortality rate	Mortality rate (%), Mortality rate (per patient or person year), Mortality rate (absolute number of deaths)
Mean/median survival	Mean/median survival (age years), free text
Life expectancy data	Life expectancy (age years), free text
Study quality	High, Moderate, Low, Very low

SCD, sickle cell disease.

Table S4. Synthesis of regional prevalence of overall SCD and specific genotypes.

S4. a. Prevalence of SCD: Africa.

**SCD prevalence in adults (age ≥18 years)**								
**Study**	**Setting**	**Country**	**Prevalence as reported in publication**	**Number of people with genotype/total population studied**	**Years of data collection**	**Male/ female (%)**	**Quality rating of study**	**Included in prevalence meta-analysis**
Zohoun 2020[1]	Military recruits	Benin	HbSC, 0.2%	HbSC, 3/1,483	2016	97.7/2.3	Moderate	Yes
Diallo 2018[2]	Routine screening of pregnant women and their newborns as part of prenatal care	Mali	SCD/β-thalassemia,0.08%<br>HbSC, 1.12%<br>HbSS, 0.21%	Sickle/β-thalassemia, 2/2,240<br>HbSC, 27/2,240<br>HbSS, 5/2,420	2010–2012	0/100	High	Yes
Burnham-Marusich 2016[3]	Nested cohort study of expectant parents	Nigeria	HbSS, 1.0%	HbSS, 42/3,371	2013–2014	0/100	Moderate	Yes
**SCD prevalence in children (age <18 years)**								
**Study**	**Setting**	**Country**	**Prevalence as reported in publication**	**Number of people with genotype/total population studied**	**Years of data collection**	**Male/ female (%)**	**Quality rating of study**	**Included in prevalence meta-analysis**
Moez 2016[4]	Screening of primary school children in an oasis, aged 6–12 years	Egypt	HbSS, 1.1%	HbSS, 4/349	NR	66.7/43.3	Low	No
Suchdev 2014[5]	Random selection of children aged 6–35 months	Kenya	HbSS, 1.6%	HbSS, 14/854	2010	50.3/49.7	Moderate	No
Adam 2019[6]	Cross-sectional study of hospitalized children	Sudan	HbSS, 3.5%	HbSS, 14/400	2017–2018	56.2/43.8	Low	No
Smart 2019[7]	Tanzania Sickle Surveillance Study (TS3), children aged ≤24 months born to HIV-infected mothers	Tanzania	GND, 1.2%	GND, NR/17,204	2017–2018	NR	Very low	No
Vovor 2014[8]	School population aged 6–9 years from a single township	Togo	HbSC, 1.92%	HbSC, 9/570	2010	NR	Low	No
Okwi 2010[9]	Random selection of children aged 6 months to 5 years	Uganda	HbSS, 1.28%	HbSS, 11/857	2007–2009	NR	High	No
Chunda-Liyoka 2018[10]	General population screening, children aged 3 months to 18 years	Zambia	GND, 3.4%	GND, 17/503	2017	NR	Very low	No
**SCD prevalence in mixed age populations**								

Study	Setting	Country	Prevalence as reported in publication	Number of people with genotype/total population studied	Years of data collection	Male/ female (%)	Quality rating of study	Included in prevalence meta-analysis
Adewoyin 2019[11]	Samples tested at the national reference lab	Nigeria	HbSC, 0.9%<br>HbSS, 13.3%	HbSC, NR/6,851<br>HbSS, NR/6,851	2016–2017	NR	Very low	No
Umoh 2010[12]	Samples tested at a university hospital	Nigeria	HbSC, 0.04%<br>HbSS, 1.50%	HbSC, 4/8,097<br>HbSS, 121/8,097	2003–2007	14.2/85.8	Very low	No
Ameen 2016[13]	Questionnaire of university students	Nigeria	HbSC, 1.3%<br>HbSS, 6.2%	HbSC, NR/372<br>HbSS, NR/372	2015	NR	Very low	No
Daak 2016[14]	Cross-section of the general population	Sudan	HbSS, 1.8%	HbSS, 16/822	2014	NR	Low	No

GND, genotype not defined; NR, not reported; SCD, sickle cell disease.

S4. b. Prevalence of SCD: Europe.

SCD prevalence in adults (age ≥18 years)								
**Study**	**Setting**	**Country**	**Prevalence as reported in publication**	**Number of people with genotype/total population studied**	**Years of data collection**	**Male/ female (%)**	**Quality rating of study**	**Included in prevalence meta-analysis**
Mueller 2015[15]	Screening of Ghanian migrants	Germany	HbSC, 0.9%<br>HbSS, 0.35%	HbSC, 5/567<br>HbSS, 2/567	2013–2014	53/47	Very low	No
Leleu 2021[16]	French nationwide claims database	France	GND, 48.6/100,000	GND, 257/703,261	2006–2016	35.4/64.6	NR	Yes
Arica 2012[17]	Premarital screening	Turkey	HbSS, 0.4%	HbSS, 631/175,600	2004–2009	50/50	High	Yes
Pinto 2019[18]	Survival rates and causes of death in elderly patients	Italy	NR	Sickle β-thalassemia, 288/NR	2015–2018	50/50	Moderate	No
**SCD prevalence in children (age <18 years)**								
**Study**	**Setting**	**Country**	**Prevalence as reported in publication**	**Number of people with genotype/total population studied**	**Years of data collection**	**Male/ female (%)**	**Quality rating of study**	**Included in prevalence meta-analysis**
Gibbons 2015[19]	Targeted screening program	Ireland	GND, 2.0%	GND, 396/19,423	2009–2012	NR	Low	No
**SCD prevalence in mixed age populations**								
**Study**	**Setting**	**Country**	**Prevalence as reported in publication**	**Number of people with genotype/total population studied**	**Years of data collection**	**Male/ female (%)**	**Quality rating of study**	**Included in prevalence meta-analysis**
Hansen 2020[20]	Danish Health registry	Denmark	2.70/100,000	GND, NR/5,707,251	2015	49.7/51.3	High	Yes
Brizido 2018[21]	Retrospective review of	Portugal	HbSC, 0.02%<br>HbSS, 0.37%	HbSC, 4/19,086<br>HbSS, 70/19,086	2011–2016	NR	Very low	No

	screening/diagnostic results							
Hemminki 2015[22]	Swedish Hospital Discharge Register	Sweden	GND, 0.0003%	GND, 584/NR	1987–2010	NR	High	No
Oktay 2016[23]	Hospital chart review	Turkey	GND, 0.01% HbSS, 0.09%	GND, 10/70,266 HbSS, 60/70,266	NR	48/52	Very low	No
Kjellander 2021[24]	Estimated through national population-based registries	Sweden	GND, 6.55/100,000	NR	2018	NR	High	Yes
Dormandy 2018[25]	Joint analysis of relevant national databases	UK	GND, 0.02% (1/4,600)	GND, 14,000/NR	2016	NR	Moderate	Yes

GND, genotype not defined; NR, not reported; SCD, sickle cell disease.

S4. c. Prevalence of SCD: India.

**SCD prevalence in adults (age ≥18 years)**								
**Study**	**Setting**	**Country**	**Prevalence as reported in publication**	**Number of people with genotype/total population studied**	**Years of data collection**	**Male/ female (%)**	**Quality rating of study**	**Included in prevalence meta-analysis**
Bhukhanvala 2012[26]	Analysis of samples collected at four communities in Surat City	India	GND, 0.06%	GND, 21/34,364	NR	53/47	Moderate	Yes
**SCD prevalence in children (age <18 years)**								
**Study**	**Setting**	**Country**	**Prevalence as reported in publication**	**Number of people with genotype/total population studied**	**Years of data collection**	**Male/ female (%)**	**Quality rating of study**	**Included in prevalence meta-analysis**
Chourasia 2020[27]	Screening of tribal school populations	India	GND, 0.7%	GND, 29/3,992	2015–2017	47/53	High	Yes
Gunjal Sandeep 2012[28]	Screening at two tribal schools	India	HbSS, 0.2%	HbSS, 2/908	2009	NR	Low	No
Oberoi 2020[29]	Screening at a tribal school, children aged 10–14 years	India	HbSC, 0.5%	HbSC, 1/211	NR	NR	Very low	No
**SCD prevalence in mixed age populations**								
**Study**	**Setting**	**Country**	**Prevalence as reported in publication**	**Number of people with genotype/total population studied**	**Years of data collection**	**Male/ female (%)**	**Quality rating of study**	**Included in prevalence meta-analysis**
Madhubala 2020[30]	Retrospective analysis of laboratory samples	India	GND, 0.12% SCD/β-thalassemia 0.12%	GND, 2/1,668 SCD/β-thalassemia, 2/1,668	2017–2020	25/75	Low	No
Maji 2020[31]	Local population screening	India	HbSS, 0.04%	HbSS, 125/287,258	2010–2020	NR	Moderate	Yes

			SCD/β-thalassemia, 0.05%	SCD/β-thalassemia, 155/287,258				
Mohanty 2013[32]	Screening of college students and pregnant women	India	SCD/β-thalassemia, 0.02%<br>HbSS, 0.04%	SCD/β-thalassemia, 9/56,780<br>HbSS, 22/56,780	2000–2005	NR	Moderate	Yes
Nakajima 2020[33]	Local Tharu population screening	Nepal	GND, 0.25%	GND, 10/4,029	2015–2019	NR	Very low	No
Panigrahi 2015[34]	Local population screening	India	GND, 0.70% (0.66)	GND, 97/15,701	2009–2011	50.7/49.3	High	Yes
Patel 2012[35]	Screening of school and college students	India	GND, 0.03%	GND, 10/32,857	2007 (end date not specified)	NR	High	Yes
Patel 2013[36]	Local population screening	India	HbSS, 1.5%	HbSS, 112/7,304	NR	NR	Moderate	No
Purohit 2014[37]	Local population screening	India	HbSC, 10.1%<br>HbSS, 3.0%	HbSC, 60/594<br>HbSS, 18/594	NR	49.2/50.8	Very low	No
Teli 2016[38]	Screening of anemic patients	India	SCD/β-thalassemia, 0.42%	SCD/β-thalassemia, 5/1,200	2012–2015	NR	Very low	No
Sidhu 2019[39]	Local Tharu population screening	Nepal	GND, 0.24%	GND, 9/3,825	2015–2018	NR	Very low	No
Shrestha 2020[40]	Retrospective analysis of laboratory samples	Nepal	SCD/β-thalassemia, 3.11%<br>HbSS, 2.39%	SCD/β-thalassemia, 125/4,018<br>HbSS, 96/4,018	2019	45/55	Very low	No

GND, genotype not defined; NR, not reported; SCD, sickle cell disease.

S4. d. Prevalence of SCD: Middle East.

**Study**	**Setting**	**Country**	**Prevalence as reported in publication**	**Number of people with genotype/total population studied**	**Years of data collection**	**Male/ female (%)**	**Quality rating of study**	**Included in prevalence meta-analysis**
**SCD prevalence in adults (age ≥18 years)**								
Al-Allawi 2010[41]	Premarital screening	Iraq	Sickle β-thalassemia, 0.08%	Sickle β-thalassemia, 1/1,182	2006	50/50	Moderate	Yes
Alsaeed 2018[42]	Premarital screening	Saudi Arabia	GND, 0.4%	GND, 4.632/1,230,332	2011–2015	50/50	Moderate	No
Memish 2011[43]	Premarital screening	Saudi Arabia	HbSS, 0.3% (0.27)	HbSS, 4,313/1,572,140	2004–2009	NR	High	Yes
Rouh AlDeen 2021[44]	Premarital screening	Kuwait	GND, 0.062%	GND, 172/275,819	2009–2020	49/51	High	Yes
**SCD prevalence in children (age <18 years)**								
**Study**	**Setting**	**Country**	**Prevalence as reported in publication**	**Number of people with genotype/total**	**Years of data collection**	**Male/ female (%)**	**Quality rating of study**	**Included in prevalenc**

				population studied				e meta-analysis
Al Arrayed 2011[45]	Annually repeated, cross-sectional study of secondary school students	Bahrain	HbSS, 1.3%	HbSS, 676/60,424	1999–2008	55/45	High	Yes
**SCD prevalence in mixed age populations**								
**Study**	**Setting**	**Country**	**Prevalence as reported in publication**	**Number of people with genotype/total population studied**	**Years of data collection**	**Male/ female (%)**	**Quality rating of study**	**Included in prevalenc e meta-analysis**
Elsayid 2015[46]	Retrospective chart review of anemic patients	Saudi Arabia	GND, 8.9%<br>HbSS, 7.2%	GND, 297/3,332<br>HbSS, 241/3,332	2011–2013	NR	Low	No

GND, genotype not defined; NR, not reported; SCD, sickle cell disease.

S4. e. Prevalence of SCD: North America.

**SCD prevalence in adults (age ≥18 years)**								
**Study**	**Setting**	**Country**	**Prevalence as reported in publication**	**Number of people with genotype/total population studied**	**Years of data collection**	**Male/ female (%)**	**Quality rating of study**	**Included in prevalence meta-analysis**
Alayed 2014[47]	HCUP-NIS–based study of SCD in pregnant women	USA	GND, 0.05%	GND, 4,262/8,821,321	1999–2008	0/100	Very low	No
**SCD prevalence in mixed age populations**								
**Study**	**Setting**	**Country**	**Prevalence as reported in publication**	**Number of people with genotype/total population studied**	**Years of data collection**	**Male/ female (%)**	**Quality rating of study**	**Included in prevalence meta-analysis**
Nalbandian 2017[48]	Retrospective review of hospital discharge sheets	Grenada	GND, 0.149%	NR	2007–2013	NR	High	No

GND, genotype not defined; HCUP-NIS, Healthcare Cost and Utilization Project Nationwide Inpatient Sample; NR, not reported; SCD, sickle cell disease.

S4. f. Prevalence of SCD: South America and the Caribbean*.

**SCD prevalence in mixed age populations**								
**Study**	**Setting**	**Country**	**Prevalence as reported in publication**	**Number of people with genotype/total population studied**	**Years of data collection**	**Male/ female (%)**	**Quality rating of study**	**Included in prevalence meta-analysis**
Pinto 2020[49]	Burden of disease model	Brazil	GND, 23.9/100,000	NR	2018	NR	Moderate	No

Santiago 2017[50]	Local population screening	Brazil	HbSC, 2.71%<br>HbSS, 0.42%	HbSC, 77/2,843<br>HbSS, 12/2,843	2010–2011	52.3/47.7	Low	No
Ilozue 2010[51]	Capture–recapture study of SCD patients in the city of Aracaju	Brazil	HbSS, 0.1%	HbSS, 400/536,785	1987–2008	51.6/48.4	Low	Yes
Lippi 2020[52]	Global Burden of Disease Study	Global	GND, 0.042%	GND, 3,140,000/NR	1997–2017	NR	High	No

*Including global. GND, genotype not defined; NR, not reported; SCD, sickle cell disease.

Table S5. Synthesis of regional prevalence of SCT (HbAS).

S5. a. Prevalence of SCT (HbAS): Africa.

SCT prevalence in adults (age ≥18 years)								
**Study**	**Setting**	**Country**	**Prevalence as reported in publication**	**Number of people with genotype/total population studied**	**Years of data collection**	**Male/ female (%)**	**Quality rating of study**	**Included in prevalenc e meta-analysis**
Zohoun 2020[1]	Military recruits	Benin	16.1%	238/1,483	2016	97.7/2.3	Moderate	Yes
Okocha 2016[53]	Cross-sectional study of apparently healthy children aged 6–24 months and their parents	Nigeria	13.4% (parents)	22/164	NR	NR	Low	No
Burnham-Marusich 2016[3]	Nested cohort study of expectant parents	Nigeria	22.0%	746/3,371	2013–2014	NR	Moderate	Yes
Diallo 2018[2]	Routine screening of pregnant women and their newborns as part of prenatal care	Mali	12.69%	307/2,420 (maternal results)	2010–2012	0/100	High	Yes
**SCT prevalence in children (age <18 years)**								
**Study**	**Setting**	**Country**	**Prevalence as reported in publication**	**Number of people with genotype/total population studied**	**Years of data collection**	**Male/ female (%)**	**Quality rating of study**	**Included in prevalenc e meta-analysis**
Moez 2016[4]	Screening of primary school children in an oasis, aged 6–12 years	Egypt	18.0%	64/349	NR	66.7/33.3	Low	No
Suchdev 2014[5]	Random selection of children aged 6–35 months	Kenya	17.1%	146/854	2010	50.3/49.7	Moderate	No
Okocha 2016[53]	Cross-sectional study of apparently healthy children aged 6–24 months and their parents	Nigeria	22.0%	18/82	NR	NR	Low	No

Adam 2019[6]	Cross-sectional study of hospitalized children	Sudan	11.3%	45/400	2017–2018	56.2/43.8	Moderate	No
Smart 2019[7]	Tanzania Sickle Surveillance Study (TS3), children aged ≤24 months born to HIV-infected mothers	Tanzania	20.3%	NR/17,204	2017–2018	NR	Very low	201
Ambrose 2020[54]	Repurposed samples for HIV testing from children aged 0–24 months	Tanzania	20.3%	3,492/17,200	2017–2018	NR	Moderate	No
Vovor 2014[8]	School population aged 6–9 years from a single township	Togo	11.93%	68/570	2010	NR	Low	No
Okwi 2010[8]	Random selection of children aged 6 months to 5 years	Uganda	10.27%	88/857	2007–2009	NR	High	No
Chunda-Liyoka 2018[10]	General population screening, children aged 3 months to 18 years	Zambia	15.5%	78/503	2017	NR	Very low	No
**SCT prevalence in mixed age populations**								
**Study**	**Setting**	**Country**	**Prevalence as reported in publication**	**Number of people with genotype/total population studied**	**Years of data collection**	**Male/ female (%)**	**Quality rating of study**	**Included in prevalence meta-analysis**
Délicat-Loembet 2014[55]	Cross-section of the general population	Gabon	21.1%	895/4,249	2005–2008	NR	High	Yes
Adewoyin 2019[11]	Samples tested at the national reference lab	Nigeria	23.3%	NR/6,851	2016–2017	NR	Very low	No
Umoh 2010[12]	Samples tested at a university hospital	Nigeria	19.6%	1,580/8,097	2003–2007	14.2/85.8	Very low	No
Ameen 2016[13]	Questionnaire of university students	Nigeria	32.7%	NR/372	2015	NR	Very low	No
Daak 2016[14]	Cross-section of the general population	Sudan	24.9%	220/882	2014	NR	Low	No

NR, not reported; SCT, sickle cell trait.

S5. b. Prevalence of SCT (HbAS): Europe.

**SCT prevalence in adults (age ≥18 years)**								
**Study**	**Setting**	**Country**	**Prevalence as reported in publication**	**Number of people with genotype/total population studied**	**Years of data collection**	**Male/ female (%)**	**Quality rating of study**	**Included in prevalence meta-analysis**
Mueller 2015[15]	Screening of Ghanian migrants	Germany	17.4%	100/576	2013–2014	53/47	Very low	No

Arica 2012[17]	Premarital screening	Turkey	3.458%	6,074/175,660	2004–2009	50/50	High	Yes
Uysal 2013[56]	Premarital screening	Turkey	GND, 0.33%	GND, 128/38,544	2011–2012	NR	High	Yes
**SCT prevalence in mixed age populations**								
**Study**	**Setting**	**Country**	**Prevalence**	**Number of people with genotype/total population studied**	**Years of data collection**	**Male/ female (%)**	**Quality rating of study**	**Included in prevalenc e meta-analysis**
Hansen 2020[20]	Danish Health registry	Denmark	8.11/100,000	NR/5,707,251	2015	49.7/51.3	High	Yes
Theodori dou 2018[57]	National Thalassaemia and SCD Prevention Programme	Greece	1.6%	541/33,837	2001–2015	NR	High	No
Brizido 2018[21]	Retrospective review of screening/diagnostic results	Portugal	3.8%	733/19,086	2011–2016	NR	Very low	No
Oktay 2016[23]	Hospital chart review	Turkey	6.30%	4,424/70,226	NR	48/52	Very low	No

NR, not reported; SCT, sickle cell trait.

S5. c. Prevalence of SCT (HbAS): India.

**SCT prevalence in adults (age ≥18 years)**								
**Study**	**Setting**	**Country**	**Prevalence as reported in publication**	**Number of people with genotype/total population studied**	**Years of data collection**	**Male/ female (%)**	**Quality rating of study**	**Included in prevalenc e meta-analysis**
Bhukhanv ala 2012[26]	Four selected communities and general population controls	India	1.66%	571/34,364	NR	53/47	Moder ate	Yes
Patel 2021[58]	Screening at university level	India	1.4%	59/4,197	2018	73.2/26.8	Moder ate	Yes
**SCT prevalence in children (age <18 years)**								
**Study**	**Setting**	**Country**	**Prevalence as reported in publication**	**Number of people with genotype/total population studied**	**Years of data collection**	**Male/ female (%)**	**Quality rating of study**	**Included in prevalenc e meta-analysis**
Chourasia 2020[27]	Screening of tribal school populations	India	14.4%	576/3,992	2015–2017	47/53	High	Yes
Gunjal Sandeep 2012[28]	Screening at two tribal schools	India	6.1%	55/908	2009	NR	Low	No
Italia 2015[59]	Newborn screening and follow-up program	India	12.57%	687/5,467	NR (over 2 years)	NR	Moder ate	No

	for sickle cell disease among South Gujarat (India) tribal populations							

SCT prevalence in mixed age populations								
**Study**	**Setting**	**Country**	**Prevalence as reported in publication**	**Number of people with genotype/total population studied**	**Years of data collection**	**Male/ female (%)**	**Quality rating of study**	**Included in prevalence meta-analysis**
Nagar 2015[60]	Screening of a population in East Indian states	India	3.4%	56/1,592	2015	NR	Moderate	Yes
Shah 2012[61]	Local population screening	India	25.99%	150/577	NR	48.9/51.1	Low	No
Patel 2013[36]	Local population screening	India	23.7%	1,740/7,307	NR	NR	Moderate	No
Patel 2012[35]	Screening of school and college students	India	1.3%	435/32,857	2007 (end date not specified)	NR	High	Yes
Dolai 2012[62]	Local population screening	India	1.1%	399/35,413	2007–2011	64/36	High	Yes
Panigrahi 2015[34]	Local population screening	India	10.6%	1,672/15,701	2009–2011	50.7/49.3	High	Yes
Maji 2020[31]	Local population screening	India	0.77%	2,203/287,258	2010–2020	NR	Moderate	Yes
Mohanty 2013[32]	Screening of college students and pregnant women	India	0.7%	396/56,780	2000–2005	Ratio 1:12	Moderate	Yes
Nakajima 2020[33]	Local population	Nepal	4.6%	184/4,029	2015–2019	NR	Very low	No
Shrestha 2020[40]	Retrospective analysis of laboratory samples	Nepal	10.9%	438/4,018	2019	45/55	Very low	No

NR, not reported; SCT, sickle cell trait.

S5. d. Prevalence of SCT (HbAS): Middle East.

SCT prevalence in adults (age ≥18 years)								
**Study**	**Setting**	**Country**	**Prevalence as reported in publication**	**Number of people with genotype/total population studied**	**Years of data collection**	**Male/ female (%)**	**Quality rating of study**	**Included in prevalence meta-analysis**
All-Allawi 2010[41]	Premarital screening	Iraq	1.1%	13/1,182	2006	50/50	Moderate	Yes
Petry 2020[63]	National survey	Oman	5.3%	NR/1,109	2016–2017	NR	Moderate	Yes
Alsaeed 2018[42]	Premarital screening	Saudi Arabia	4.58%	4,632/1,230,332	2011–2015	50/50	Moderate	No
Memish 2011[43]	Premarital screening	Saudi Arabia	4.2%	66,649/1,572,140	2004–2009	NR	High	Yes

Rouh AlDeen 2021[44]	Premarital screening	Kuwait	1.81%	5,003/275,819	2009–2020	49/51	High	Yes
Mir 2020[64]	Premarital screening	Saudi Arabia	0.32%	12/3,755	2016–2019	52.01/ 47.99	High	Yes
**SCT prevalence in children (age <18 years)**								
**Study**	**Setting**	**Country**	**Prevalence as reported in publication**	**Number of people with genotype/total population studied**	**Years of data collection**	**Male/ female (%)**	**Quality rating of study**	**Included in prevalence meta-analysis**
Al-Alawi 2014[65]	Follow-up testing of anemic infants	Bahrain	NR	25/1,080	2012	NR	Moderate	No
Al Arrayed 2011[45]	Annually repeated, cross-sectional study of secondary school students	Bahrain	13.3%	7,257/60,424	1999–2008	55/45	High	Yes
Petry 2020[63]	National survey	Oman	5.3%	NR/1,109	2016–2017	NR	Moderate	Yes
**SCT prevalence in mixed age populations**								
**Study**	**Setting**	**Country**	**Prevalence as reported in publication**	**Number of people with genotype/total population studied**	**Years of data collection**	**Male/ female (%)**	**Quality rating of study**	**Included in prevalence meta-analysis**
El Ariss 2016[66]	Retrospective general population cohort	Lebanon	0.49%	899/184,105	2002–2014	35/65	High	No

NR, not reported; SCD, sickle cell disease.

S5. e. Prevalence of SCT (HbAS): South America and the Caribbean.

**SCT prevalence in adults (age ≥18 years)**								
**Study**	**Setting**	**Country**	**Prevalence as reported in publication**	**Number of people with genotype/total population studied**	**Years of data collection**	**Male/ female (%)**	**Quality rating of study**	**Included in prevalence meta-analysis**
Rosenfeld 2019[67]	Brazilian National Health Survey	Brazil	2.49%	234/8,715	2014–2015	NR	High	Yes
Kroger 2022[68]	Screening of blood donors	Brazil	1.77%	127/7,166	2008	49.61/ 50.39	Moderate	Yes
**SCT prevalence in mixed age populations**								
**Study**	**Setting**	**Country**	**Prevalence as reported in publication**	**Number of people with genotype/total population studied**	**Years of data collection**	**Male/ female (%)**	**Quality rating of study**	**Included in prevalence meta-analysis**
De Assis 2015[69]	Local population screening	Brazil	2.0%	7/318	2009	NR	High	No
Santiago 2017[50]	Local population screening	Brazil	4.57%	130/2,843	2010–2011	52.3/47.7	Low	No

Marcheco-Teruel 2019[70]	Screening of pregnant women as part of the Sickle Cell Anemia Prevention Program in Cuba	Cuba	3.5%	168,865/4,847,239	1982–2018	0/100	Low		No

NR, not reported; SCT, sickle cell trait.

Table S6. Synthesis of regional birth prevalence of overall SCD and specific genotypes.

S6. a. Birth prevalence of SCD: Africa.

SCD birth prevalence in children (age <18 years)								
**Study**	**Setting**	**Country**	**Birth prevalence as reported in publication**	**Birth prevalence (cases/total population studied)**	**Years of data collection**	**Male/ female (%)**	**Quality rating of study**	**Included in birth prevalence meta-analysis**
McGann 2013[71]	A prospective newborn screening and treatment program for sickle cell anemia	Angola	HbSC, 0.02%<br>HbSS, 1.51%	HbSC, 7/36,453<br>HbSS, 550/36,453	2011–2013	NR	Moderate	Yes
Kreuels 2010[72]	Cohort study of children aged 2–4 months at enrolment	Ghana	HbSC, 1.2%<br>HbSS, 0.6%	HbSC 12/1,010<br>HbSS, 6/1,010	2003–2005	NR	Moderate	Yes
Segbefia 2019[73]	Newborn screening for SCD	Ghana	GND, 1.8%	79/4,527	2017–2018	NR	Moderate	Yes
Uyoga 2019[74]	Prospective cohort study of children with and without SCD	Kenya	β-thalassemia, 0.01%<br>HbSS, 0.08%	β-thalassemia, 10/15,702<br>HbSS, 118/15,702	2006–2011	NR	High	Yes
Nnodu 2020[75]	Implementing newborn screening for SCD as part of immunization programs in Nigeria	Nigeria	HbSC, 0.1%<br>HbSS,1.4%	HbSC, 4/3,603<br>HbSS, 51/3,603	2017–2019	50/50	High	Yes
Diallo 2018[2]	Routine screening of pregnant women and their newborns as part of prenatal care	Mali	HbSC, 0.4%<br>HbSS, 0.24%	HbSC, 10/2,489<br>HbSS, 6/2,489	2010–2012	NR	High	Yes
Nkya[76] 2019	Newborn screening for SCD	Tanzania	GND, 0.8%	GND, 31/3,981	2015–2016	50.7/49.3	Moderate	Yes
Ambrose 2020[54]	Repurposed samples for HIV testing from children aged 0–24 months	Tanzania	GND, 1.2%	GND, 210/17,200	2017–2018	NR	Moderate	Yes
Hernandez 2021[77]	Specific SCD screening and joint SCD/HIV screening for children aged ≤24 months	Uganda	GND, 2.8%	NR/278,585	2014–2019	50/50	High	Yes

Tshilolo 2009[78]	Neonatal screening for sickle cell anemia in the Democratic Republic of the Congo	Congo	HbSS, 1.37%	HbSS, 428/31,204	NR	50/50	High	Yes
Eastburg 2020[79]	Extremely high birth prevalence of SCD in rural Tanzania	Tanzania	GND, 3.9%	GND, 39/999	2019	NR	Moderate	No

GND, genotype not defined; HIV, human immunodeficiency virus; NR, not reported; SCD, sickle cell disease.

S6. b. Birth prevalence of SCD: Europe.

**SCD birth prevalence in children (age <18 years)**								
**Study**	**Setting**	**Country**	**Birth prevalence as reported in publication**	**Birth prevalence (cases/total population studied)**	**Years of data collection**	**Male/ female (%)**	**Quality rating of study**	**Included in birth prevalence meta-analysis**
Gulbis 2018[80]	Neonatal screening for SCD in Belgium for more than 20 years	Belgium	NR	GND, 251/358,331	2009–2017	NR	High	Yes
Lobitz 2014[81]	Incidence of SCD in an unselected cohort of neonates born in Berlin	Germany	NR	HbSC, 4/34,084<br>HbSS, 10/34,084	2011–2012	NR	High	Yes
Kunz 2016[82]	Significant prevalence of SCD in Southwest Germany: results from a birth cohort study	Germany	Sickle β-thalassemia, 0.01%	Sickle β-thalassemia, 3/37,838	2012–2013	NR	High	Yes
Grosse 2015[83]	The prevalence of SCD and its implication for newborn screening in Germany	Germany	NR	HbSC, 5/16,697<br>HbSS, 3/16,697	2013–2014	NR	High	Yes
Lobitz 2019[84]	Newborn screening by tandem mass spectrometry confirms the high prevalence of SCD among German newborns	Germany	NR	HbSC, 3/29,079<br>HbSS, 4/29,079	2015–2016	NR	High	Yes
Martella 2019[85]	Evaluation of technical issues in a pilot multicenter newborn screening program for SCD	Italy	HbSC, 0.02%<br>HbSS, 0.055%	HbSC, 1/5,439<br>HbSS, 3/5,439	2016–2017	NR	High	Yes
Cela 2017[86]	National registry of hemoglobinopathies in Spain (REPHem)	Spain	NR	Sickle β^0 thalassemia, 28/426,303<br>HbSC, 64/426,303<br>HbSS, 497/426,303	2014–2015	NR	Moderate	Yes

Lo Riso 2018[87]	Newborn screening for SCD: experience in Balearic Islands	Spain	NR	HbSS, 1/6,399	2016–2018	NR	Moderate	Yes
Garcia-Morin 2020[88]	Newborn SCD screening	Spain	NR	HbSS, 151/1,048,222 Sickle β^0 thalassemia, 6/1,048,222	2003–2018	NR	High	Yes
Delgado-Pecellin 2020[89]	Results of the neonatal screening on Western Andalusia	Spain	NR	Sickle β-thalassemia, 1/55,576 HbSC, 1/55,576	2018–2019	NR	High	Yes
Streetly 2018[90]	Evaluation of newborn sickle cell screening program	UK	GND, 0.039%	GND, 1,317/NR	2010–2015	NR	High	Yes

GND, genotype not defined; NR, not reported; SCD, sickle cell disease.

S6. c. Birth prevalence of SCD: India.

**SCD birth prevalence in children (age <18 years)**								
**Study**	**Setting**	**Country**	**Birth prevalence as reported in publication**	**Birth prevalence (cases/total population studied)**	**Years of data collection**	**Male/ female (%)**	**Quality rating of study**	**Included in birth prevalence meta-analysis**
Italia 2015[59]	Newborn screening and follow-up program for SCD among South Gujarat (India) tribal populations	India	HbSS, 0.6%	HbSS, 33/5,467	NR (over 2 years)	NR	Moderate	Yes

NR, not reported; SCD, sickle cell disease.

S6. d. Birth prevalence of SCD: Middle East.

**SCD birth prevalence in children (age <18 years)**								
**Study**	**Setting**	**Country**	**Birth prevalence as reported in publication**	**Birth prevalence (cases/total population studied)**	**Years of data collection**	**Male/ female (%)**	**Quality rating of study**	**Included in birth prevalence meta-analysis**
Al-Alawi 2014[65]	Follow-up testing of anemic infants	Bahrain	GND, 0.4	GND, 5/1,230	2012	NR	Moderate	Yes
Al Arrayed, Al Hajeri 2012[91]	Newborn screening services in Bahrain between 1985 and 2010	Bahrain	NR	GND, 228/38,940	2007–2010	NR	Moderate	Yes
Al-Alawi, Sarhan 2014[65]	Prevalence of anemia among 9-month-old infants attending primary care in Bahrain	Bahrain	NR	GND, 7/1.080	2012	NR	Moderate	Yes

Khoriaty 2014[92]	Incidence of SCD and other hemoglobin variants in Lebanese neonates	Lebanon	NR	Sickle β^0 thalassemia, 2/10,095<br>HbSC, 1/10,095<br>HbSS, 10/10,095	2010–2013	50.2/49.8	Moderate	Yes
Alkindi 2010[93]	Neonatal screening in Omani neonates	Oman	HbSS, 0.3%	HbSS, 19/6,317	2005–2007	NR	Moderate	Yes
Al Hosani 2014[94]	Comprehensive national neonatal screening	UAE	NR	Sickle β-thalassemia, 28/542,286<br>HbSS, 203/542,286	2002–2011	NR	High	Yes

GND, genotype not defined; NR, not reported; SCD, sickle cell disease.

S6. e. Birth prevalence of SCD: North America.

SCD birth prevalence in children (age <18 years)								
**Study**	**Setting**	**Country**	**Birth prevalence as reported in publication**	**Birth prevalence (cases/total population studied)**	**Years of data collection**	**Male/ female (%)**	**Quality rating of study**	**Included in birth prevalence meta-analysis**
Zhou 2021[95]	The Alberta newborn screening approach for SCD	Canada	NR	Sickle β-thalassemia, 0.1/10,000<br>HbSC, 1.1/10,000<br>HbSS, 3/10,000	2019–2020	NR	High	Yes
Feuchtbaum 2012[96]	Birth prevalence of disorders detectable through newborn screening by race/ethnicity	USA	NR	Sickle β-thalassemia, 2.5/100,000<br>Sickle β^0 thalassemia, 0.6/100,000<br>HbSC, 6.2/100,000<br>HbSS, 10.6/100,000	2005–2010	NR	High	Yes
Therrell 2015[97]	Newborn screening for SCDs in the United States	USA	NR	Sickle β-thalassemia, 0.043/1,000<br>HbSC, 0.164/1,000<br>HbSS, 0.314/1,000	1991–2010	NR	High	Yes
Smeltzer 2018[98]	Birth prevalence of sickle cell trait and SCD in Shelby County	USA	GND, 0.2%	GND, 324/158,616	2002–2012	NR	High	Yes
Wang 2015[99]	Mortality of New York children with SCD identified through newborn screening	USA	NR	HbSS and HbS-β^0 thalassemia combined: 1,173/2,200,000	2000–2008	NR	High	No

GND, genotype not defined; NR, not reported; SCD, sickle cell disease.

S6. f. Birth prevalence of SCD: South America and the Caribbean.

SCD birth prevalence in children (age <18 years)								
**Study**	**Setting**	**Country**	**Birth prevalence as reported in publication**	**Birth prevalence (cases/total population studied)**	**Years of data collection**	**Male/ female (%)**	**Quality rating of study**	**Included in birth prevalence meta-analysis**
Wagner 2010[100]	Neonatal screening for hemoglobinopathies: in a public health system	Brazil	Sickle β-thalassemia, 0.001%<br>HbSC, 0.001%<br>HbSS, 0.01%	Sickle β-thalassemia, 7/437,787<br>HbSC, 7/437,788<br>HbSS, 33/437,787	2004–2007	NR	High	Yes
de Castro Lobo 2014[101]	Newborn screening program for hemoglobinopathies	Brazil	NR	Sickle β-thalassemia, 3/100,000<br>HbSC, 16/100,000<br>HbSS, 52/100,000	2001–2010	NR	Low	Yes
Sabarense 2015[102]	Comprehensive newborn screening program	Brazil	NR	Sickle β^0 thalassemia, 92/3,617,919<br>HbSC, 1,014/3,617,919<br>GND, 1,451/3,617,919	1998–2002	NR	Moderate	Yes
Carlos 2015[103]	Hemoglobinopathies in newborns in the southern region of the Triângulo Mineiro, Brazil	Brazil	NR	HbSS, 2/1,004	2011–2013	49.9/50.1	High	Yes
Silva 2016[104]	Screening for structural hemoglobin variants in Bahia, Brazil	Brazil	HbSC, 0.13%<br>HbSS, 0.18%	HbSS, 20/14,773<br>HbSC, 27/14,773	2006–2009	NR	High	Yes
Eller, da Silva 2016[105]	Evaluation of a neonatal screening program for SCD	Brazil	NR	Sickle β-thalassemia, 26/730,069<br>HbSC, 12/730,069	2003–2012	NR	High	Yes
Knight-Madden 2019[106]	Newborn screening for SCD in the Caribbean	French Guiana	HbSC, 0.16%<br>HbSS, 0.25%	HbSC, 186/115,200<br>HbSS, 293/115,200	1984–2013	NR	High	Yes
Knight-Madden 2019[106]	Newborn screening for SCD in the Caribbean	Grenada	HbSC, 0.1%<br>HbSS, 0.52%	HbSC, 2/1,914<br>HbSS, 10/1,914	2014–2015	NR	High	Yes
Knight-Madden 2019[106]	Newborn screening for SCD in the Caribbean	Guadeloupe	HbSC, 0.13%<br>HbSS, 0.17%	HbSC, 231/178,428<br>HbSS, 310/178,428	1984–2010	NR	High	Yes

Alladin 2022[107]	Newborn screening for sickle cell anemia and congenital hypothyroidism	Guyana	GND, 0.34%	GND, 7/2,039	2016–2017	NR	Moderate to low	Yes
Rotz 2013[108]	Prevalence of SCD, hemoglobin S, and hemoglobin C among Haitian newborns	Haiti	HbSC, 0.13%<br>HbSS, 0.44%	HbSC, 3/2,258<br>HbSS, 10/2,258	2010	NR	High	Yes
Alvarez 2019[109]	Newborn screening for SCD using point-of-care testing	Haiti	NR	Sickle β-thalassemia, 11/2,149<br>HbSC, 5/2,149<br>HbSS, 16/2,149	2017–2018	53/47	Moderate	Yes
Serjeant 2017[110]	Newborn screening for SCD in Jamaica	Jamaica	Sickle β^0 thalassemia, 0.009%<br>Sickle β-thalassemia, 0.052%<br>HbSC, 0.221%<br>HbSS, 0.305%	Sickle β^0 thalassemia, 6/66,833<br>Sickle β-thalassemia, 35/66,833<br>HbSC, 148/66,833<br>HbSS, 204/66,833	2008–2015	NR	High	Yes
Knight-Madden 2019[106]	Newborn screening for SCD in the Caribbean	Jamaica	HbSC, 0.23%<br>HbSS, 0.41%	HbSC, 95/40,044<br>HbSS, 165/40,444	2016–2017	NR	High	Yes
Knight-Madden 2019[106]	Newborn screening for SCD in the Caribbean	Martinique	HbSC, 0.1%<br>HbSS, 0.15%	HbSC, 29/30,171<br>HbSS, 44/30,171	2009–2015	NR	High	Yes
Knight-Madden 2019[106]	Newborn screening for SCD in the Caribbean	St Lucia	HbSC, 0.25%<br>HbSS, 0.15%	HbSC, 5/2,023<br>HbSS, 3/2,038	2015–2017	NR	High	Yes
Knight-Madden 2019[106]	Newborn screening for SCD in the Caribbean	Tobago	HbSC, 0.19%<br>HbSS, 0.38%	HbSC, 14/7,389<br>HbSS, 28/7,389	2008–2017	NR	High	Yes

GND, genotype not defined; NR, not reported; SCD, sickle cell disease.

Table S7. Synthesis of regional birth prevalence of SCT (HbAS).

S7. a. Birth prevalence of SCT (HbAS): Africa.

SCT birth prevalence in children (age <18 years)								
**Study**	**Setting**	**Country**	**Birth prevalence as reported in publication**	**Birth prevalence (cases/total population studied)**	**Years of data collection**	**Male/ female (%)**	**Quality rating of study**	**Included in birth prevalence meta-analysis**
McGann 2013[71]	Newborn screening and treatment program for sickle cell anemia	Angola	21.0%	7,666/36,453	2011–2013	NR	Moderate	Yes

Dokekias 2022[111]	Neonatal screening for sickle cell disease	Congo	NR	558/2,897	2019–2020	Sex ratio F/M 1.03	NR	Yes
Kreuels 2010[72]	Cohort study of children aged 2–4 months at enrolment	Ghana	10.9%	110/1,010	2003–2005	NR	Moderate	Yes
Hernandez 2021[77]	Specific SCD and joint SCD/HIV screening for children ≤24 months	Uganda	14.7%	NR/278,585	2014–2019	50/50	High	Yes
Diallo 2018[2]	Routine screening of pregnant women and their newborns as part of prenatal care	Mali	7.96%	198/2,489 (newborn results)	2010–2012	NR	High	Yes
Nnodu 2020[75]	Implementing newborn screening for SCD as part of immunization programs	Nigeria	20.5%	740/3,603	2017–2019	50/50	High	Yes
Tshilolo 2009[78]	Neonatal screening for sickle cell anemia	Congo	16.9%	5,276/31,204	NR	50/50	High	Yes
Nkya 2019[76]	Newborn screening for SCD	Tanzania	12.7%	505/3,981	2015–2016	50.7/49.3	Moderate	Yes
Ambrose 2020[54]	Repurposed samples for HIV testing from children aged 0–24 months	Tanzania	20.3%	3,492/17,200	50	NR	Moderate	Yes
Eastburg 2020[79]	Extremely high birth prevalence of SCD in rural Tanzania	Tanzania	31.6%	315/999	2019	NR	Moderate	No

HIV, human immunodeficiency virus; NR, not reported; SCD, sickle cell disease; SCT, sickle cell trait.

S7. b. Birth prevalence of SCT (HbAS): Europe.

**SCT birth prevalence in children (age <18 years)**								
**Study**	**Setting**	**Country**	**Birth prevalence as reported in publication**	**Birth prevalence (cases/total population studied)**	**Years of data collection**	**Male/ female (%)**	**Quality rating of study**	**Included in birth prevalence meta-analysis**
Gulbis 2018[80]	Neonatal screening for sickle cell disease in Belgium for more than 20 years	Belgium	NR	5,727/358,331	2009–2017	NR	High	Yes
Kunz 2016[82]	Significant prevalence of sickle cell disease in Southwest Germany: results from a birth cohort study	Germany	0.23%	86/37,838	2012–2013	NR	High	Yes
Lobitz 2014[81]	Incidence of sickle cell disease in an unselected cohort of	Germany	NR	265/34,084	2011–2012	NR	High	Yes

	neonates born in Berlin							
Grosse 2016[83]	The prevalence of sickle cell disease and its implication for newborn screening in Germany	Germany	NR	98/16,697	2013–2014	NR	High	Yes
Lobitz 2019[84]	Newborn screening by tandem mass spectrometry confirms the high prevalence of sickle cell disease among German newborns	Germany	NR	139/29,079	2015–2016	NR	High	Yes
Martella 2018[85]	Pilot multicenter newborn screening program for sickle cell disease	Italy	0.68%	37/5,439	2016–2017	NR	High	Yes
Lo Riso 2018[87]	Newborn screening for sickle cell disease: experience in Balearic Islands	Spain	NR	79/19,197	2016–2018	NR	Moderate	Yes
Delgado-Pecellin 2020[89]	Results of the neonatal screening on Western Andalusia after a decade of experience	Spain	NR	128/55,576	2018–2019	NR	High	Yes

NR, not reported; SCT, sickle cell trait.

S7. c. Birth prevalence of SCT (HbAS): India.

SCT birth prevalence in children (age <18 years)								
**Study**	**Setting**	**Country**	**Birth prevalence as reported in publication**	**Birth prevalence (cases/total population studied)**	**Years of data collection**	**Male/ female (%)**	**Quality rating of study**	**Included in birth prevalence meta-analysis**
Italia 2015[59]	Newborn screening and follow-up program for sickle cell disease among South Gujarat (India) tribal populations	India	12.5%	689/5,467	NR (over 2 years)	NR	Moder ate	Yes

NR, not reported; SCT, sickle cell trait.

S7. d. Birth prevalence of SCT (HbAS): Middle East.

SCT birth prevalence in children (age <18 years)								
**Study**	**Setting**	**Country**	**Birth prevalence as reported in publication**	**Birth prevalence (cases/total population studied)**	**Years of data collection**	**Male/ female (%)**	**Quality rating of study**	**Included in birth prevalence meta-analysis**

Al-Alawi, Sarhan 2014[65]	Prevalence of anemia among 9-month-old infants attending primary care in Bahrain	Bahrain	NR	35/1,230	2012	NR	Moderate	Yes
Khoriaty 2014[92]	Incidence of sickle cell disease and other hemoglobin variants in Lebanese neonates	Lebanon	NR	166/10,095	2010–2013	50.2/49.8	Moderate	Yes
Alkindi 2010[93]	Neonatal screening in Omani neonates	Oman	4.8%	303/6,317	2005–2007	NR	Moderate	Yes
Al Hosani 2014[94]	Comprehensive national neonatal screening	UAE	NR	4,481/542,286	2002–2011	NR	High	Yes

NR, not reported; SCT, sickle cell trait.

S7. e. Birth prevalence of SCT (HbAS): North America.

SCT birth prevalence in children (age <18 years)								
**Study**	**Setting**	**Country**	**Birth prevalence as reported in publication**	**Birth prevalence (cases/ total population studied)**	**Years of data collection**	**Male/ female (%)**	**Quality rating of study**	**Included in birth prevalence meta-analysis**
Zhou 2021[95]	The Alberta newborn screening approach for sickle cell disease	Canada	NR	75.7/10,000	2019–2020	NR	High	Yes
Ojudu 2014[112]	Incidence of sickle cell trait, United States	USA	1.55%	55,258/3,576,297	2010	NR	High	Yes
Smeltzer 2016[98]	Birth prevalence of SCT and sickle cell disease in Shelby County	USA	4.16%	6,606/158,616	2002–2012	NR	High	Yes
Gbadamosi-Akindele 2019[113]	A state-level retrospective analysis of newborn screening for hemoglobinopathies	USA	NR	161.4/10,000	2011–2018	NR	High	Yes

NR, not reported; SCT, sickle cell trait.

S7. f. Prevalence of SCT (HbAS): South America and the Caribbean.

SCT prevalence in children (age <18 years)								
**Study**	**Setting**	**Country**	**Birth prevalence as reported in publication**	**Birth prevalence (cases/total population studied)**	**Years of data collection**	**Male/ female (%)**	**Quality rating of study**	**Included in birth prevalence meta-analysis**
Wagner 2010[100]	Neonatal screening for hemoglobinopathie	Brazil	1.19%	5,236/437,787	2004–2007	NR	High	Yes

	s: in a public health system							
de Castro Lobo 2014[101]	Newborn screening program for hemoglobinopathies	Brazil	4,060/100,000	49,424/1,217,833	2001–2010	NR	High	Yes
Carlos 2015[103]	Hemoglobinopathies in newborns in the southern region of the Triângulo Mineiro, Brazil.	Brazil	NR	46/1,004	2011–2013	49.9/50.1	High	Yes
Silva 2016[104]	Screening for structural hemoglobin variants in Bahia, Brazil	Brazil	5.8%	861/14,773	2006–2009	NR	High	Yes
Eller, da Silva 2016[105]	Evaluation of a neonatal screening program for sickle cell disease	Brazil	NR	6,173/730,069	2003–2012	NR	High	Yes
Knight-Madden 2019[106]	Newborn screening for sickle cell disease in the Caribbean	French Guiana	7.66%	8,824/115,200	1992–2013	NR	High	Yes
Knight-Madden 2019[106]	Newborn screening for sickle cell disease in the Caribbean	Grenada	9.56%	183/1.914	2014–2015	NR	High	Yes
Knight-Madden 2019[106]	Newborn screening for sickle cell disease in the Caribbean	Guadeloupe	7.92%	14,126/178,428	1984–2010	NR	High	Yes
Alladin 2022[107]	Newborn screening for sickle cell anemia and congenital hypothyroidism	Guyana	8.41%	172/2,039	2016–2017	NR	Moderate to low	Yes
Rotz 2013[108]	Prevalence of sickle cell disease, hemoglobin S, and hemoglobin C among Haitian newborns	Haiti	2.5%	247/2,258	2010	NR	High	Yes
Alvarez 2019[109]	Newborn screening for sickle cell disease using point-of-care testing	Haiti	13.94%	301/2,149	2017–2018	53/47	Moderate	Yes
Serjeant 2017[110]	Newborn screening for sickle cell disease in Jamaica	Jamaica	9.81%	6,559/66,833	2008–2015	NR	High	Yes

Knight-Madden 2019[106]	Newborn screening for sickle cell disease in the Caribbean	Jamaica	9.94%	4,020/40,444	2016–2017	NR	High	Yes
Knight-Madden 2019[106]	Newborn screening for sickle cell disease in the Caribbean	Martinique	7.07%	2,134/30,171	2009–2015	NR	High	Yes
Knight-Madden 2019[106]	Newborn screening for sickle cell disease in the Caribbean	St Lucia	11.76%	238/2,023	2015–2017	NR	High	Yes
Knight-Madden 2019[106]	Newborn screening for sickle cell disease in the Caribbean	Tobago	9.32%	689/7,389	2008–2017	NR	High	Yes

NR, not reported; SCT, sickle cell trait.

Table S8. Causes of death among people with sickle cell disease.

Publication		Maria-Pinto 2019,[18] Italy	Telfer 2018,[114] UK	Bardón Cancho 2020,[115] Spain	Brousse 2019,[116] France	Desselas 2020,[117] France	Gualandro 2015,[118] Brazil	Serjeant 2018,[110] Jamaica	Streetly 2018,[90] UK	Rettenbacher 2021,[119] Netherlands	Total
**Number of deaths**		**59**	**10**	**18**	**18**	**75**	**14**	**119**	**9**	**23**	**345**
**Cause of death**		N (%)									
Vaso-occlusive crisis										3 (13.0)	3 (0.9)
Acute chest syndrome		19 (32.2)	2 (20.0)	1 (5.6)		6 (8.0)	1 (7.1)	31 (26.1)		1 (4.3)	61 (17.7)
Splenic sequestration				3 (16.7)	2 (11.1)	9 (12.0)		12 (10.1)	2 (22.2)		28 (8.1)
Pulmonary hypertension		1 (1.7)								1 (4.3)	2 (0.6)
Stroke		7 (11.9)	2 (20.0)	1 (5.6)		3 (4.0)	2 (14.3)	11 (9.2)			26 (7.5)
Thromboembolic events	Pulmonary embolism					1 (1.3)	1 (7.1)				2 (0.6)
	Cerebrovascular accident									5 (21.7)	5 (1.4)
Heart failure/other cardiac causes			1 (10.0)		1 (5.6)		1 (7.1)				3 (0.9)
Infection	Sepsis or septicemia	2 (3.4)		3 (16.7)	6 (33.3)			23 (19.3)			34 (9.9)
	Other infection		2 (20.0)	1 (5.6)	2 (11.1)	21 (28.0)	2 (14.3)	3 (2.5)	1 (11.1)	8 (34.8)	40 (11.6)
Anemia						6 (8.0)					6 (1.7)
Cerebral hemorrhage			1 (10.0)			3 (4.0)					4 (1.2)
Congenital disorders	Congenital encephalopathy			1 (5.6)							1 (0.3)
	Other congenital disorders			1 (5.6)							1 (0.3)
	Renal failure	2 (3.4)					1 (7.1)	1 (0.8)			4 (1.2)

Organ failure hepatic failure	15 (25.4)		1 (5.6)			1 (7.1)			2 (8.7)	19 (5.5)
Malignancy	8 (13.6)		1 (5.6)			1 (7.1)				10 (2.9)
Neurological							3 (2.5)			3 (0.9)
Post-transplant complication			3 (16.7)							3 (0.9)
Pregnancy-associated			1 (5.6)			1 (7.1)		3 (33.3)		5 (1.4)
Other/not known	5 (8.5)	2 (20.2)	1 (5.6)	7 (38.9)	26 (34.7)	3 (21.4)	35 (29.4)	3 (33.3)	3 (13.0)	85 (24.6)

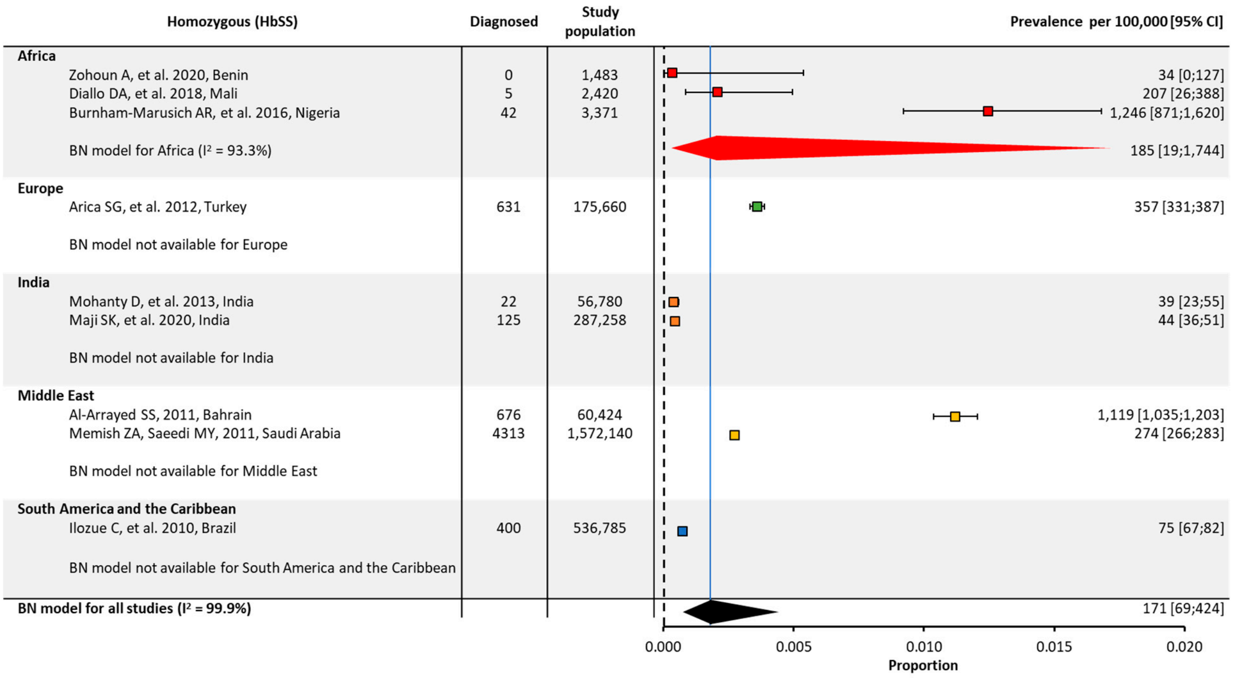

Figure S1. Global and regional prevalence* of homozygous SCD (HbSS). Africa Africa,[1-3] Europe,[17] India,[31,32] Middle East.[45,51]. *Within each region the prevalence is estimated using a binomial normal model, which assumes a binomial distribution for the individual studies with a mean value drawn from a normal distribution for a regional/global value. A summary estimate is determined for each region with >2 studies. North America had insufficient data to determine the prevalence of SCD. BN, binomial normal; CI, confidence interval; SCD, sickle cell disease.

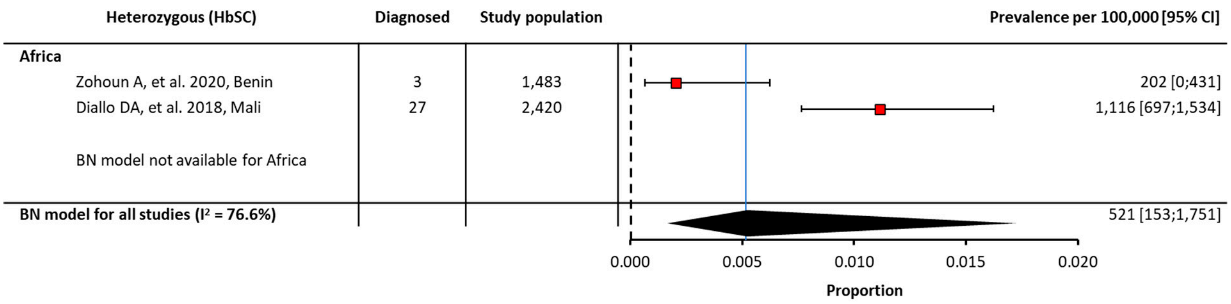

Figure S2. Global and regional prevalence* of heterozygous SCD (HbSC). Africa.[1,2]. *Within each region the prevalence is estimated using a binomial normal model, which assumes a binomial distribution for the individual studies with a mean value drawn from a normal distribution for a regional/global value. A summary estimate is determined for each region with >2 studies. North America had insufficient data to determine the prevalence of SCD. BN, binomial normal; CI, confidence interval; SCD, sickle cell disease.

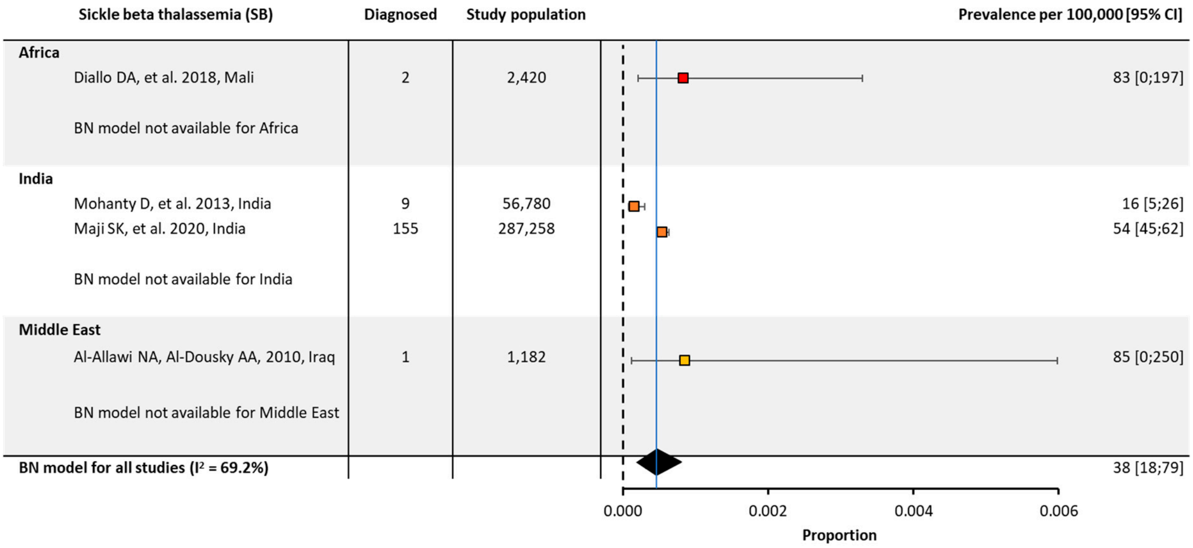

Figure S3. Global and regional prevalence* of sickle cell β-thalassemia. Africa,[2] India,[31,32] Middle East.[41]. *Within each region the prevalence is estimated using a binomial normal model, which assumes a binomial distribution for the individual studies with a mean value drawn from a normal distribution for a regional/global value. A summary estimate is determined for each region with >2 studies. North America had insufficient data to determine the prevalence of SCD. BN, binomial normal; CI, confidence interval; SCD, sickle cell disease.

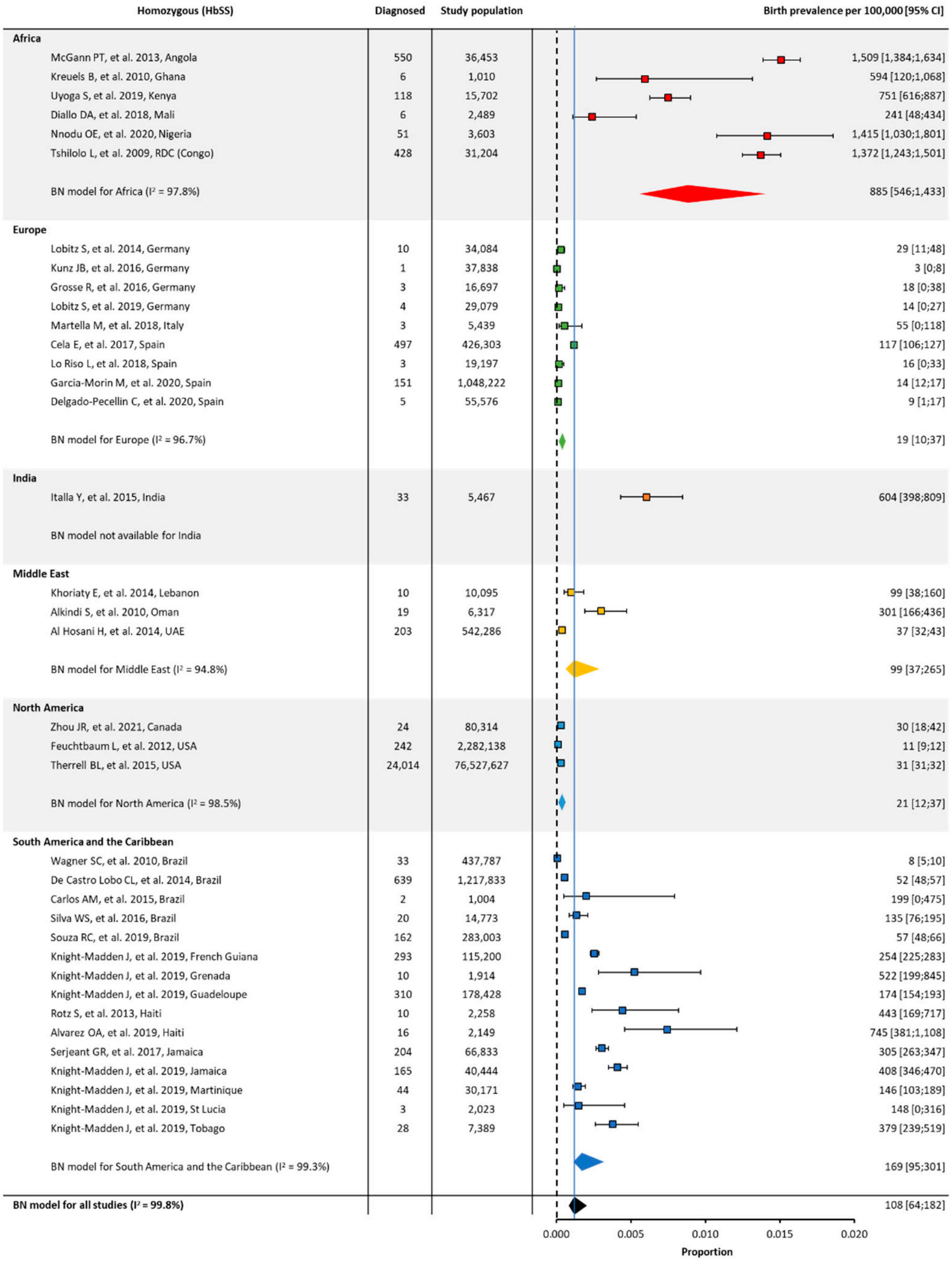

Figure S4. Global and regional birth prevalence* of homozygous SCD (HbSS). Africa,[2,71,72,74,75,78] Europe,[81-89] India,[59] Middle East,[92-94] North America,[95-97] South America and Caribbean.[100,101,103,104,106,108-110,120]. *Within each region the birth prevalence is estimated using a binomial-normal model, which assumes a binomial distribution for the individual studies with a mean value drawn from a normal distribution for a regional/global value. A summary estimate is determined for each region with >2 studies. North America had insufficient data to determine the prevalence of SCD. BN, binomial normal; CI, confidence interval; SCD, sickle cell disease.

Heterozygous (HbSC)	Diagnosed	Study population	Birth prevalence per 100,000 [95% CI]
**Africa**			
McGann PT, et al. 2013, Angola	7	36,453	19 [5;33]
Kreuels B, et al. 2010, Ghana	12	1,010	1,188 [520;1,856]
Diallo DA, et al. 2018, Mali	10	2,489	402 [153;650]
Nnodu OE, et al. 2020, Nigeria	4	3,603	111 [2;220]
BN model for Africa (I^2 = 94.8%)			171 [37;791]
**Europe**			
Lobitz S, et al. 2014, Germany	4	34,084	12 [0;23]
Grosse R, et al. 2016, Germany	5	16,697	30 [4;56]
Lobitz S, et al. 2019, Germany	3	29,079	10 [0;22]
Martella M, et al. 2018, Italy	1	5,439	18 [0;54]
Cela E, et al. 2017, Spain	64	426,303	15 [11;19]
Garcia-Morin M, et al. 2020, Spain	27	1,048,222	3 [2;4]
Delgado-Pecellin C, et al. 2020, Spain	1	55,576	2 [0;5]
BN model for Europe (I^2 = 86.4%)			4 [4;17]
**Middle East**			
Khoriaty E, et al. 2014, Lebanon	1	10,095	10 [0;29]
BN model not available for Middle East			
**North America**			
Zhou JR, et al. 2021, Canada	9	80,314	11 [4;19]
Feuchtbaum L, et al. 2012, USA	141	2,282,138	6 [5;7]
Therrell BL, et al. 2015, USA	12,382	76,527,627	16 [16;16]
BN model for North America (I^2 = 96.3%)			10 [6;17]
**South America and the Caribbean**			
Wagner SC, et al. 2010, Brazil	7	437,787	2 [0.4;3]
De Castro Lobo CL, et al. 2014, Brazil	201	1,217,833	17 [14;19]
Sabarense AP, et al. 2015, Brazil	1,014	3,617,919	28 [26;30]
Silva WS, et al. 2016, Brazil	27	14,773	183 [114;252]
Eller R, da Silva DB, 2016, Brazil	12	730,069	2 [0.7;3]
Knight-Madden J, et al. 2019, French Guiana	186	115,200	161 [138;185]
Knight-Madden J, et al. 2019, Grenada	2	1,914	104 [0;249]
Knight-Madden J, et al. 2019, Guadeloupe	231	178,428	129 [113;146]
Rotz S, et al. 2013, Haiti	3	2,258	133 [0;283]
Alvarez OA, et al. 2019, Haiti	5	2,149	233 [29;436]
Serjeant GR, et al. 2017, Jamaica	148	66,833	221 [186;257]
Knight-Madden J, et al. 2019, Jamaica	95	40,444	235 [188;282]
Knight-Madden J, et al. 2019, Martinique	29	30,171	96 [61;131]
Knight-Madden J, et al. 2019, St Lucia	5	2,023	247 [31;464]
Knight-Madden J, et al. 2019, Tobago	14	7,389	189 [90;289]
BN model for South America and the Caribbean (I^2 = 99.6%)			65 [28;150]
**BN model for all studies (I^2 = 99.8%)**			36 [19;67]

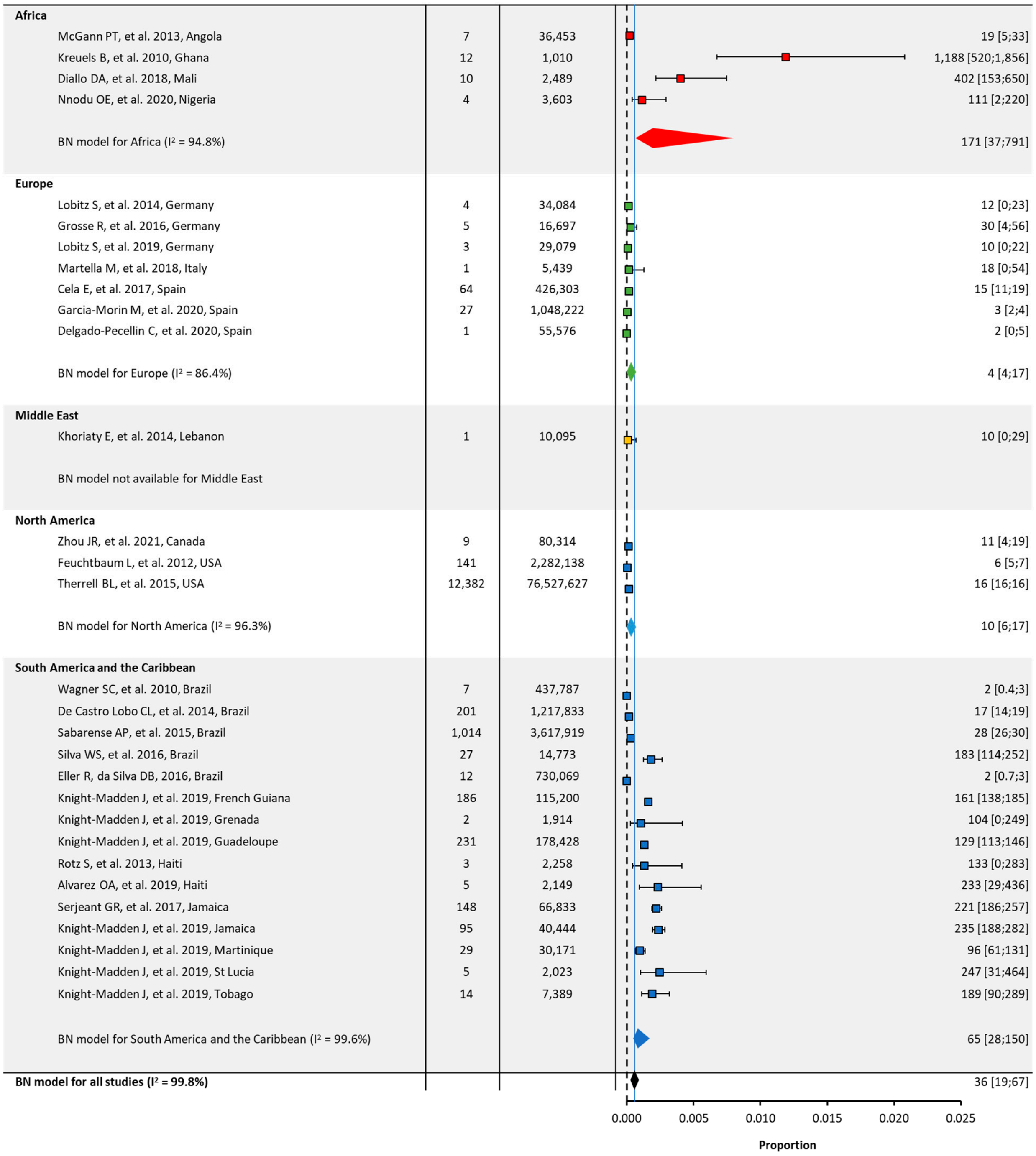

Figure S5. Global and regional birth prevalence* of heterozygous SCD (HbSC). Africa,[2,71,72,75] Europe,[81,83-86,88,89] Middle East,[92] North America,[95-97] South America and the Caribbean.[100-102,104-106,108-110]. *Within each region the birth prevalence is estimated using a binomial normal model, which assumes a binomial distribution for the individual studies with a mean value drawn from a normal distribution for a regional/global value. A summary estimate is determined for each region with >2 studies. North America had insufficient data to determine the prevalence of SCD. BN, binomial normal; CI, confidence interval; SCD, sickle cell disease.

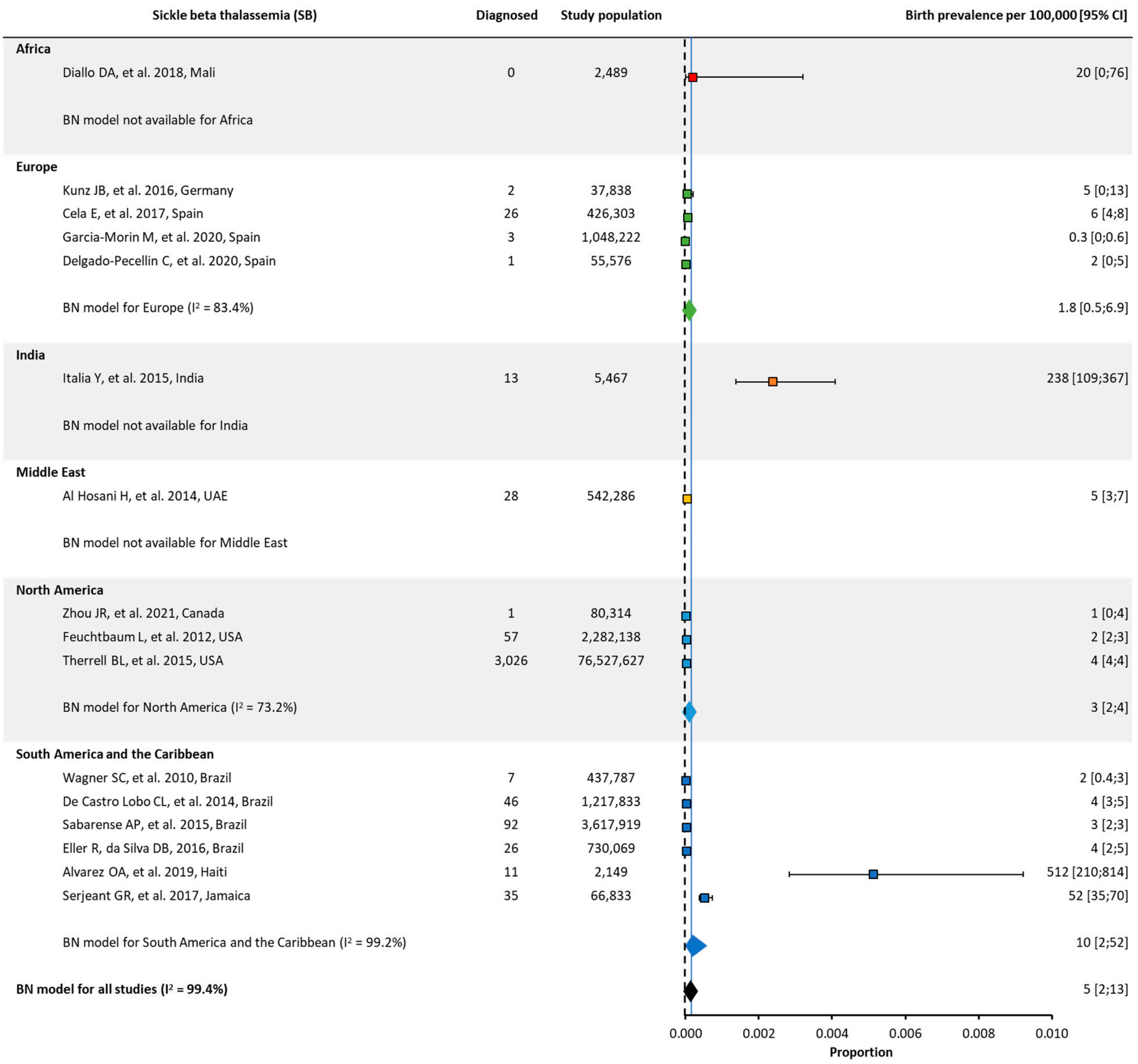

Figure S6. Global and regional birth prevalence* of sickle cell β-thalassemia. Africa,[2]·[82,86,88] Europe,[82,86,88,89] India,[59] Middle East,[94] North America,[95-97] South America and the Caribbean.[100-102,105,109,110]. *Within each region the birth prevalence is estimated using a binomial-normal model, which assumes a binomial distribution for the individual studies with a mean value drawn from a normal distribution for a regional/global value. A summary estimate is determined for each region with >2 studies. North America had insufficient data to determine the prevalence of SCD. BN, binomial normal; CI, confidence interval; SCD, sickle cell disease.

References

1. Zohoun, A.; Baglo Agbodande, T.; Zohoun, L.; Anani, L. Prevalence of hemoglobin abnormalities in an apparently healthy population in Benin. *Hematol Transfus Cell Ther* **2020**, *42*, 145-149.
2. Diallo, D.A.; Guindo, A.; Toure, B.A.; Sarro, Y.S.; Sima, M.; Tessougue, O.; Baraika, M.A.; Guindo, P.; Traore, M.; Diallo, M.; et al. [Targeted newborn screening for sickle-cell anemia: Sickling test (Emmel test) boundaries in the prenatal assessment in West African area]. *Rev Epidemiol Sante Publique* **2018**, *66*, 181-185.
3. Burnham-Marusich, A.; Ezeanolue, C.; Obiefune, M.; Yang, W.; Osuji, A.; Ogidi, A.; Hunt, A.; D., P.; Ezeanolue, E.E. Prevalence Of Sickle Cell Trait And Reliability Of Self-Reported Status Among Expectant Parents In Nigeria: Implications For Targeted Newborn Screening. *Public Health Genomics* **2016**, *19*.
4. Moez, P.; Younan, D.N. High prevalence of haemoglobin S in the closed Egyptian community of Siwa Oasis. *J Clin Pathol* **2016**, *69*, 632-636.

5. Suchdev, P.S.; Ruth, L.J.; Earley, M.; Macharia, A.; Williams, T.N. The burden and consequences of inherited blood disorders among young children in western Kenya. *Matern Child Nutr* **2014**, *10*, 135-144.
6. Adam, M.A.; Adam, N.K.; Mohamed, B.A. Prevalence of sickle cell disease and sickle cell trait among children admitted to Al Fashir Teaching Hospital North Darfur State, Sudan. *BMC Res Notes* **2019**, *12*, 659.
7. Smart, L.; Ambrose, E.; Charles, M.; Hernandez, A.; Latham, T.; Hokororo, A.; Beyanga, M.; Kamugisha, E.; Tebuka, E.; Howard, T.; et al. Genetic Analysis in the Tanzania Sickle Surveillance Study (TS3): Modifiers of Sickle Cell Disease and Identification of Hemoglobin Variants. *Blood* **2019**, *134*, 988.
8. Vovor, A.; Feteke, L.; Kueviakoe, I.M.; Kpatarou, L.; Mawussi, K.; Magnang, H.; Segbena, A.Y. Blood typing profile of a school-aged population of a North Togo township. *Hemoglobin* **2014**, *38*, 316-318.
9. Okwi, A.L.; Byarugaba, W.; Ndugwa, C.M.; Parkes, A.; Ocaido, M.; Tumwine, J.K. An up-date on the prevalence of sickle cell trait in Eastern and Western Uganda. *BMC Blood Disord* **2010**, *10*, 5.
10. Chunda-Liyoka, C.; Kumar, A.A.; Sambo, P.; Lubinda, F.; Nchimba, L.; Humpton, T.; Okuku, P.; Miyanda, C.; Im, J.; Maguire, K.; et al. Application of a public health strategy to large-scale point-of-care screening for sickle cell disease in rural sub-Saharan Africa. *Blood Adv* **2018**, *2*, 1-3.
11. Adewoyin, A.; Busari, O.; Aworanti, O. Hemoglobin phenotypes in Nigeria: Data from a national reference laboratory. *American Journal of Clinical Pathology* **2019**, *152*, S115-S116.
12. Umoh, A.V.; Abah, G.M.; Ekanem, T.I.; Essien, E.M. Haemoglobin genotypes: a prevalence study and implications for reproductive health in Uyo, Nigeria. *Niger J Med* **2010**, *19*, 36-41.
13. Ameen, H.; Abidoye, A.; Alatishe-Muhammad, B.; Aderibigbe, S.; Uthman, M.; Bolarinwa, O.; Saludeen, A.; Musa, O.; Akande, T. Prevalence of heamoglobin genotype screening and awareness of SCD among undergraduate students of Unilorin. *Journal of Medicine and Biomedical Research:* **2016**, 14-27.
14. Daak, A.A.; Elsamani, E.; Ali, E.H.; Mohamed, F.A.; Abdel-Rahman, M.E.; Elderdery, A.Y.; Talbot, O.; Kraft, P.; Ghebremeskel, K.; Elbashir, M.I.; et al. Sickle cell disease in western Sudan: genetic epidemiology and predictors of knowledge attitude and practices. *Trop Med Int Health* **2016**, *21*, 642-653.
15. Muller, S.A.; Amoah, S.K.; Meese, S.; Spranger, J.; Mockenhaupt, F.P. High prevalence of anaemia among African migrants in Germany persists after exclusion of iron deficiency and erythrocyte polymorphisms. *Trop Med Int Health* **2015**, *20*, 1180-1189.
16. Leleu, H.; Arlet, J.B.; Habibi, A.; Etienne-Julan, M.; Khellaf, M.; Adjibi, Y.; Pirenne, F.; Pitel, M.; Granghaud, A.; Sinniah, C.; et al. Epidemiology and disease burden of sickle cell disease in France: A descriptive study based on a French nationwide claim database. *PLoS One* **2021**, *16*, e0253986.
17. Arica, S.; Turhan, E.; Özer, C.; Arica, V.; Silfeler, D.; Silfeler, I.; Altun, A. Evaluation of hemoglobinopathy screening results of a six year period in Turkey. *International Journal of Collaborative Research on Internal Medicine & Public Health* **2012**.
18. Pinto, V.M.; Graziadei, G.; Voi, V.; Quota, A.; Rigano, P.; Spadola, V.; Fidone, C.; Gianesin, B.; De Franceschi, L.; Forni, G.L. Survival rates and causes of death in elderly patients with sickle cell disease. *HemaSphere* **2019**, *3*, 322-323.
19. Gibbons, C.; Geoghegan, R.; Conroy, H.; Lippacott, S.; O'Brien, D.; Lynam, P.; Langabeer, L.; Cotter, M.; Smith, O.; McMahon, C. Sickle cell disease: time for a targeted neonatal screening programme. *Ir Med J* **2015**, *108*, 43-45.
20. Hansen, D.L.; Glenthoj, A.; Moller, S.; Biemond, B.J.; Andersen, K.; Gaist, D.; Petersen, J.; Frederiksen, H. Prevalence of Congenital Hemolytic Disorders in Denmark, 2000-2016. *Clin Epidemiol* **2020**, *12*, 485-495.
21. Brízido, H.; Silva, C.; Sousa, M.; Ribeiro, R.; Sousa, J.; Sousa, G. Hemoglobinopathies: A Five Year Revised Experience of Centro Medicina Laboratorial Germano de Sousa. *Clin Chem Lab Med 2018; 56(2): eA1–eA29* **2018**, *56*, eA20 - eA21.
22. Hemminki, K.; Li, X.; Forsti, A.; Sundquist, J.; Sundquist, K. Thalassemia and sickle cell anemia in Swedish immigrants: Genetic diseases have become global. *SAGE Open Med* **2015**, *3*, 2050312115613097.
23. Oktay, G.; Acıpayam, C.; İlhan, G.; Karal, Y.; Sakallı, G.; Yılmazoglu, N.; Basun, S. The results of hemoglobinopathy screening in Hatay, the southern part of Turkey. *J Clin Anal Med* **2016**, *7*, 6-9.
24. Kjellander, C.; Hernlund, E.; Ivergård, M.; Svedbom, A.; Dibbern, T.; Stenling, A.; Sjöö, F.; Vertuani, S.; Glenthøj, A.; Cherif, H. Sickle Cell Disease in Sweden - Prevalence and Resource Use Estimated through Population-Based National Registers. *Blood Rev* **2021**, *138*, 2040.
25. Dormandy, E.; James, J.; Inusa, B.; Rees, D. How many people have sickle cell disease in the UK? *J Public Health (Oxf)* **2018**, *40*, e291-e295.
26. Bhukhanvala, D.S.; Sorathiya, S.M.; Shah, A.P.; Patel, A.G.; Gupte, S.C. Prevalence and hematological profile of beta-thalassemia and sickle cell anemia in four communities of Surat city. *Indian J Hum Genet* **2012**, *18*, 167-171.
27. Chourasia, S.; Kumar, R.; Singh, M.; Vishwakarma, C.; Gupta, A.K.; Shanmugam, R. High Prevalence of Anemia and Inherited Hemoglobin Disorders in Tribal Populations of Madhya Pradesh State, India. *Hemoglobin* **2020**, *44*, 391-396.
28. Gunjal, S.; Narlawar, U.; Humne, A.; Chaudhari, V. Prevalence of sickle cell disorder and anaemia in tribal school students from central India. *International Journal of Collaborative Research on Internal Medicine & Public Health* **2012**, *4*, 1321-1329.
29. Oberoi, A.; Kanakia, S. Prevalence of thalassemia syndromes, hemoglobinopathies and mutation analysis in a tribal school in India. In Proceedings of the 11th World Hematology and Oncology Congress & 47th World Congress on Nursing Care, Rome, July 24-25, 2020.
30. Madhubala, R.; Gupta, A.; Kumar, M.; Saini, S.; Purohit, A.; Didel, S.; Elhence, P.; Singh, K. Prevalence of Haemoglobinopathies in Western Rajasthan. *Indian J Hematol Blood Transfus (Nov 2020) 36(Suppl 1):S1–S229* **2020**, *36*, S82.

31. Maji, S.K.; Dolai, T.K.; Pradhan, S.; Maity, A.; Mandal, S.; Mondal, T.; Manna, S.; Mandal, P.K. Implications of Population Screening for Thalassemias and Hemoglobinopathies in Rural Areas of West Bengal, India: Report of a 10-Year Study of 287,258 Cases. *Hemoglobin* **2020**, *44*, 432-437.
32. Mohanty, D.; Colah, R.B.; Gorakshakar, A.C.; Patel, R.Z.; Master, D.C.; Mahanta, J.; Sharma, S.K.; Chaudhari, U.; Ghosh, M.; Das, S.; et al. Prevalence of beta-thalassemia and other haemoglobinopathies in six cities in India: a multicentre study. *J Community Genet* **2013**, *4*, 33-42.
33. Nakajima, L.; Dascalu, I.; Marchand, K.; Khaira, A.; Yu, S.; Xu, R. Assessment of the Sickle Cell Disease Screening Program in the Indigenous Tharu Population in Nepal. *J Investig Med* **2020**, *68(Suppl 1):A1–A228*, A178.
34. Panigrahi, S.; Patra, P.K.; Khodiar, P.K. The screening and morbidity pattern of sickle cell anemia in chhattisgarh. *Indian J Hematol Blood Transfus* **2015**, *31*, 104-109.
35. Patel, A.G.; Shah, A.P.; Sorathiya, S.M.; Gupte, S.C. Hemoglobinopathies in South Gujarat population and incidence of anemia in them. *Indian J Hum Genet* **2012**, *18*, 294-298.
36. Patel, J.; Patel, B.; Gamit, N.; Serjeant, G.R. Screening for the sickle cell gene in Gujarat, India: a village-based model. *J Community Genet* **2013**, *4*, 43-47.
37. Purohit, P.; Dehury, S.; Patel, S.; Patel, D.K. Prevalence of deletional alpha thalassemia and sickle gene in a tribal dominated malaria endemic area of eastern India. *ISRN Hematol* **2014**, *2014*, 745245.
38. Teli, A.B.; Deori, R.; Saikia, S.P.; Pathak, K.; Panyang, R.; Rajkakati, R. beta-Thalassaemia and its Co-existence with Haemoglobin E and Haemoglobin S in Upper Assam Region of North Eastern India: A Hospital Based Study. *J Clin Diagn Res* **2016**, *10*, GC01-04.
39. Sidhu, H.; Donaldson, M.; Dayan, Z.; Dean, P.; Dhinsa, J.; Marchand, N.; Wang, A.; Zou, V.; Kapoor, V. Follow-up assessment of the sickle cell disease screening program in the indigenous Tharu population of Nepal. *J Investigative Med* **2019**, *67*, 167.
40. Shrestha, R.M.; Pandit, R.; Yadav, U.K.; Das, R.; Yadav, B.K.; Upreti, H.C. Distribution of Hemoglobinopathy in Nepalese Population. *J Nepal Health Res Counc* **2020**, *18*, 52-58.
41. Al-Allawi, N.; Al-Dousky, A. Frequency of haemoglobinopathies at premarital health screening in Dohuk, Iraq: implications for a regional prevention programme. *East Mediterr Health J* **2010**, 381-385.
42. Alsaeed, E.S.; Farhat, G.N.; Assiri, A.M.; Memish, Z.; Ahmed, E.M.; Saeedi, M.Y.; Al-Dossary, M.F.; Bashawri, H. Distribution of hemoglobinopathy disorders in Saudi Arabia based on data from the premarital screening and genetic counseling program, 2011-2015. *J Epidemiol Glob Health* **2018**, *7 Suppl 1*, S41-S47.
43. Memish, Z.A.; Saeedi, M.Y. Six-year outcome of the national premarital screening and genetic counseling program for sickle cell disease and beta-thalassemia in Saudi Arabia. *Ann Saudi Med* **2011**, *31*, 229-235.
44. Rouh AlDeen, N.; Osman, A.; Alhabashi, M.; Al Khaldi, R.; Alawadi, H.; Alromh, M.; Alyafai, E.; Akbulut-Jeradi, N. The Prevalence of β-Thalassemia and Other Hemoglobinopathies in Kuwaiti Premarital Screening Program: An 11-Year Experience. *J Pers Med* **2021**, *11*, 980.
45. Al Arrayed, S. Prevalence of abnormal hemoglobins among students in Bahrain: A ten-year study. *Bahrain Med Bull* **2011**, *33*.
46. Elsayid, M.; Al-Shehri, M.J.; Alkulaibi, Y.A.; Alanazi, A.; Qureshi, S. Frequency distribution of sickle cell anemia, sickle cell trait and sickle/beta-thalassemia among anemic patients in Saudi Arabia. *J Nat Sci Biol Med* **2015**, *6*, S85-88.
47. Alayed, N.; Kezouh, A.; Oddy, L.; Abenhaim, H.A. Sickle cell disease and pregnancy outcomes: population-based study on 8.8 million births. *J Perinat Med* **2014**, *42*, 487-492.
48. Nalbandian, M.; H Kaminsky, H.; Baghdasaryan, P.; Keleny, D.; Nalbandyan, K.; Jalonen, T. Epidemiology of Sickle Cell Disease in Grenada: A comparison with Haiti, Jamaica and the United States of America. **2017**, *66*, 491-496.
49. Pinto, A.C., FF.; Gualandro, S.; Fonseca, P.; Bueno, C.; Cançado, R. Burden of sickle cell disease: A Brazilian societal perspective analysis. *Blood* **2020**, *136*, 10-11.
50. Santiago, R.P.; Oliveira, R.M.; Soares, L.F.; Figueiredo, C.V.B.; Silva, D.O.; Hurtado-Guerrero, A.F.; Fiuza, L.M.; Guarda, C.C.; Adorno, E.V.; Barbosa, C.G.; et al. Hemoglobin Variant Profiles among Brazilian Quilombola Communities. *Hemoglobin* **2017**, *41*, 83-88.
51. Ilozue, C.; Cipolotti, R.; Melo, C.A.; Gurgel, R.Q.; Cuevas, L.E. Estimating the post-neonatal prevalence of sickle cell disease in a Brazilian population. *Trop Med Int Health* **2010**, *15*, 1125-1131.
52. Lippi, G.; Mattiuzzi, C. Updated Worldwide Epidemiology of Inherited Erythrocyte Disorders. *Acta Haematol* **2020**, *143*, 196-203.
53. Okocha, C.; Onubogu, C.U.; Aneke, J.; Onah, C.; Ajuba, I.; Ibeh, N.; Egbuonu, I. Prevalence of sickle cell gene among apparently healthy under-two south-east Nigerian children: what is the role of parental premarital counselling and socio-demographic characteristics? A pilot study. *Niger J Med* **2016**, *25*, 176-181.
54. Ambrose, E.E.; Smart, L.R.; Charles, M.; Hernandez, A.G.; Latham, T.; Hokororo, A.; Beyanga, M.; Howard, T.A.; Kamugisha, E.; McElhinney, K.E.; et al. Surveillance for sickle cell disease, United Republic of Tanzania. *Bull World Health Organ* **2020**, *98*, 859-868.
55. Delicat-Loembet, L.M.; Elguero, E.; Arnathau, C.; Durand, P.; Ollomo, B.; Ossari, S.; Mezui-me-ndong, J.; Mbang Mboro, T.; Becquart, P.; Nkoghe, D.; et al. Prevalence of the sickle cell trait in Gabon: a nationwide study. *Infect Genet Evol* **2014**, *25*, 52-56.
56. Uysal, A.; Genc, A.; Tasyurek, N.; Turkyilmaz, B. Prevalence of beta-thalassemia trait and abnormal hemoglobin in premarital screening in the province of Izmir, Turkey. *Pediatr Hematol Oncol* **2013**, *30*, 46-50.

57. Theodoridou, S.; Prapas, N.; Balassopoulou, A.; Boutou, E.; Vyzantiadis, T.A.; Adamidou, D.; Delaki, E.E.; Yfanti, E.; Economou, M.; Teli, A.; et al. Efficacy of the National Thalassaemia and Sickle Cell Disease Prevention Programme in Northern Greece: 15-Year Experience, Practice and Policy Gaps for Natives and Migrants. *Hemoglobin* **2018**, *42*, 257-263.
58. Patel, G.M.; Parmar, A.; Zalavadiya, D.; Talati, K. Tackling the Menace of Anemia and Hemoglobinopathies among Young Adults - Conceptualizing University-Level Screening. *Indian J Community Med* **2021**, *46*, 117-120.
59. Italia, Y.; Krishnamurti, L.; Mehta, V.; Raicha, B.; Italia, K.; Mehta, P.; Ghosh, K.; Colah, R. Feasibility of a newborn screening and follow-up programme for sickle cell disease among South Gujarat (India) tribal populations. *J Med Screen* **2015**, *22*, 1-7.
60. Nagar, R.; Sinha, S.; Raman, R. Haemoglobinopathies in eastern Indian states: a demographic evaluation. *J Community Genet* **2015**, *6*, 1-8.
61. Shah, A.; Hussain, R.; Fareed, M.; Afzal, M. Gene frequency of sickle cell trait among Muslim populations in a malarial belt of India, i.e., Manipur. *Egyptian Journal of Medical Human Genetics*
62. Dolai, T.K.; Dutta, S.; Bhattacharyya, M.; Ghosh, M.K. Prevalence of hemoglobinopathies in rural Bengal, India. *Hemoglobin* **2012**, *36*, 57-63.
63. Petry, N.; Al-Maamary, S.A.; Woodruff, B.A.; Alghannami, S.; Al-Shammakhi, S.M.; Al-Ghammari, I.K.; Tyler, V.; Rohner, F.; Wirth, J.P. National Prevalence of Micronutrien Deficiencies, Anaemia, Genetic Blood Disorders and Over- and Undernutrition in Omani Women of Reproductive Age and Preschool Children. *Sultan Qaboos Univ Med J* **2020**, *20*, e151-e164.
64. Mir, S.A.; Alshehri, B.M.; Alaidarous, M.; Banawas, S.S.; Dukhyil, A.; Alturki, M.K. Prevalence of Hemoglobinopathies (beta-Thalassemia and Sickle Cell Trait) in the Adult Population of Al Majma'ah, Saudi Arabia. *Hemoglobin* **2020**, *44*, 47-50.
65. Al-Alawi, M.; Sarhan, N. Prevalence of anemia among nine-month-old infants attending primary care in Bahrain. *J Bahrain Med Soc* **2014**, *25*, 29-32.
66. El Ariss, A.B.; Younes, M.; Matar, J.; Berjaoui, Z. Prevalence of Sickle Cell Trait in the Southern Suburb of Beirut, Lebanon. *Mediterr J Hematol Infect Dis* **2016**, *8*, e2016015.
67. Rosenfeld, L.G.; Bacal, N.S.; Cuder, M.A.M.; Silva, A.G.D.; Machado, I.E.; Pereira, C.A.; Souza, M.F.M.; Malta, D.C. Prevalence of hemoglobinopathies in the Brazilian adult population: National Health Survey 2014-2015. *Rev Bras Epidemiol* **2019**, *22Suppl 02*, E190007 SUPL 190002.
68. Kroger, F.L.; Ernesto, I.C.; Silva, M.S.; Santos, O.F.D.; Medeiros, R.L.; Rodrigues, D.O.W. Hemoglobin S identification in blood donors: A cross section of prevalence. *Hematol Transfus Cell Ther* **2021**.
69. De Assis, E.; Araújo, J.; de Rezende, M.; da Cunha Oliveira, C.; Francisco, P.; de Melo, M. Prevalence of variant hemoglobins and thalassemias in a maroon community in Sergipe, Brazil. *Acta Scientiarum : Health Sciences* **2015**, *37*, 211-216.
70. Marcheco-Teruel, B. Sickle Cell Anemia in Cuba: Prevention and Management, 1982-2018. *MEDICC Rev* **2019**, *21*, 34-38.
71. McGann, P.T.; Ferris, M.G.; Ramamurthy, U.; Santos, B.; de Oliveira, V.; Bernardino, L.; Ware, R.E. A prospective newborn screening and treatment program for sickle cell anemia in Luanda, Angola. *Am J Hematol* **2013**, *88*, 984-989.
72. Kreuels, B.; Kreuzberg, C.; Kobbe, R.; Ayim-Akonor, M.; Apiah-Thompson, P.; Thompson, B.; Ehmen, C.; Adjei, S.; Langefeld, I.; Adjei, O.; et al. Differing effects of HbS and HbC traits on uncomplicated falciparum malaria, anemia, and child growth. *Blood* **2010**, *115*, 4551-4558.
73. Segbefia, C.; Goka, B.; Welbeck, J.; Oppong, S.; Odame, I. Implementing newborn screening for sickle cell disease in Accra, Ghana: Results and challenges. *Pediatr Blood Cancer* **2019**, *66*, 8266.
74. Uyoga, S.; Macharia, A.W.; Mochamah, G.; Ndila, C.M.; Nyutu, G.; Makale, J.; Tendwa, M.; Nyatichi, E.; Ojal, J.; Otiende, M.; et al. The epidemiology of sickle cell disease in children recruited in infancy in Kilifi, Kenya: a prospective cohort study. *Lancet Glob Health* **2019**, *7*, e1458-e1466.
75. Nnodu, O.E.; Sopekan, A.; Nnebe-Agumadu, U.; Ohiaeri, C.; Adeniran, A.; Shedul, G.; Isa, H.A.; Owolabi, O.; Chianumba, R.I.; Tanko, Y.; et al. Implementing newborn screening for sickle cell disease as part of immunisation programmes in Nigeria: a feasibility study. *Lancet Haematol* **2020**, *7*, e534-e540.
76. Nkya, S.; Mtei, L.; Soka, D.; Mdai, V.; Mwakale, P.; Mrosso, P.; Mchoropa, I.; Rwezaula, S.; Azayo, M.; Ulenga, N.; et al. Newborn screening for sickle cell disease: an innovative pilot program to improve child survival in Dar es Salaam, Tanzania. *Int Health* **2019**, *11*, 589-595.
77. Hernandez, A.G.; Kiyaga, C.; Howard, T.A.; Ssewanyana, I.; Ndeezi, G.; Aceng, J.R.; Ware, R.E. Trends in sickle cell trait and disease screening in the Republic of Uganda, 2014-2019. *Trop Med Int Health* **2021**, *26*, 23-32.
78. Tshilolo, L.; Aissi, L.M.; Lukusa, D.; Kinsiama, C.; Wembonyama, S.; Gulbis, B.; Vertongen, F. Neonatal screening for sickle cell anaemia in the Democratic Republic of the Congo: experience from a pioneer project on 31 204 newborns. *J Clin Pathol* **2009**, *62*, 35-38.
79. Eastburg, L.; Peckham, A.; Kawira, E.; Chirangi, B.; Adler, D.; Akungo, B.D.; Smart, L.R.; Ambrose, E.E. Extremely high birth prevalence of sickle cell disease in rural Tanzania. *Pediatr Blood Cancer* **2020**, *67*, e28620.
80. Gulbis, B.; Le, P.Q.; Ketelslegers, O.; Dresse, M.F.; Adam, A.S.; Cotton, F.; Boemer, F.; Bours, V.; Minon, J.M.; Ferster, A. Neonatal Screening for Sickle Cell Disease in Belgium for More than 20 Years: An Experience for Comprehensive Care Improvement. *Int J Neonatal Screen* **2018**, *4*, 37.
81. Lobitz, S.; Frommel, C.; Brose, A.; Klein, J.; Blankenstein, O. Incidence of sickle cell disease in an unselected cohort of neonates born in Berlin, Germany. *Eur J Hum Genet* **2014**, *22*, 1051-1053.
82. Kunz, J.B.; Awad, S.; Happich, M.; Muckenthaler, L.; Lindner, M.; Gramer, G.; Okun, J.G.; Hoffmann, G.F.; Bruckner, T.; Muckenthaler, M.U.; et al. Significant prevalence of sickle cell disease in Southwest Germany: results from a birth cohort study indicate the necessity for newborn screening. *Ann Hematol* **2016**, *95*, 397-402.

83. Grosse, R.; Lukacs, Z.; Cobos, P.N.; Oyen, F.; Ehmen, C.; Muntau, B.; Timmann, C.; Noack, B. The Prevalence of Sickle Cell Disease and Its Implication for Newborn Screening in Germany (Hamburg Metropolitan Area). *Pediatr Blood Cancer* **2016**, *63*, 168-170.
84. Lobitz, S.; Klein, J.; Brose, A.; Blankenstein, O.; Frömmel, C. Newborn screening by tandem mass spectrometry confirms the high prevalence of sickle cell disease among German newborns. *Ann Hematol* **2019**, *98*, 47-53.
85. Martella, M.; Viola, G.; Azzena, S.; Schiavon, S.; Biondi, A.; Basso, G.; Corti, P.; Colombatti, R.; Masera, N.; Sainati, L. Evaluation of Technical Issues in a Pilot Multicenter Newborn Screening Program for Sickle Cell Disease. *Int J Neonatal Screen* **2019**, *5*, 2.
86. Cela, E.; Bellon, J.M.; de la Cruz, M.; Belendez, C.; Berrueco, R.; Ruiz, A.; Elorza, I.; Diaz de Heredia, C.; Cervera, A.; Valles, G.; et al. National registry of hemoglobinopathies in Spain (REPHem). *Pediatr Blood Cancer* **2017**, *64*.
87. Lo Riso, L.; Ortuño Cabrero, A.; Bauza, J.; Garcia-Recio, M.; Rodriguez, B.; Andrade, B.; Sanchez-Raga, J.; Mayol, A.; Pastor, M. Newborn screening for sickle cell disease: Experience in Balearic islands 2 years after of the implementation of the screening program. In Proceedings of the 23rd European Hematology Association Congress, Stockholm, Sweden, 2018.
88. Garcia-Morin, M.; Bardon-Cancho, E.J.; Belendez, C.; Zamarro, R.; Beliz-Mendiola, C.; Gonzalez-Rivera, M.; Vecilla, C.; Llorente-Otones, L.; Perez-Alonso, V.; Roman, S.S.; et al. Fifteen years of newborn sickle cell disease screening in Madrid, Spain: an emerging disease in a European country. *Ann Hematol* **2020**, *99*, 1465-1474.
89. Delgado-Pecellin, C.; Alvarez Rios, I.; Bueno Delgado, M.D.A.; Jimenez Jambrina, M.M.; Quintana Gallego, M.E.; Ruiz Salas, P.; Marcos Luque, I.; Melguizo Madrid, E. [Results of the neonatal screening on Western Andalusia after a decade of experience.]. *Rev Esp Salud Publica* **2020**, *94*.
90. Streetly, A.; Sisodia, R.; Dick, M.; Latinovic, R.; Hounsell, K.; Dormandy, E. Evaluation of newborn sickle cell screening programme in England: 2010-2016. *Arch Dis Child* **2018**, *103*, 648-653.
91. Al Arrayed, S.; Al Hajeri, A. Newborn Screening Services in Bahrain between 1985 and 2010. *Adv Hematol* **2012**, *2012*, 903219.
92. Khoriaty, E.; Halaby, R.; Berro, M.; Sweid, A.; Abbas, H.A.; Inati, A. Incidence of sickle cell disease and other hemoglobin variants in 10,095 Lebanese neonates. *PLoS One* **2014**, *9*, e105109.
93. Alkindi, S.; Al Zadjali, S.; Al Madhani, A.; Daar, S.; Al Haddabi, H.; Al Abri, Q.; Gravell, D.; Berbar, T.; Pravin, S.; Pathare, A.; et al. Forecasting hemoglobinopathy burden through neonatal screening in Omani neonates. *Hemoglobin* **2010**, *34*, 135-144.
94. Al Hosani, H.; Salah, M.; Osman, H.M.; Farag, H.M.; El-Assiouty, L.; Saade, D.; Hertecant, J. Expanding the comprehensive national neonatal screening programme in the United Arab Emirates from 1995 to 2011. *East Mediterr Health J* **2014**, *20*, 17-23.
95. Zhou, J.R.; Ridsdale, R.; MacNeil, L.; Lilley, M.; Hoang, S.; Christian, S.; Blumenschein, P.; Wolan, V.; Bruce, A.; Singh, G.; et al. The Alberta Newborn Screening Approach for Sickle Cell Disease: The Advantages of Molecular Testing. *Int J Neonatal Screen* **2021**, *7*.
96. Feuchtbaum, L.; Carter, J.; Dowray, S.; Currier, R.J.; Lorey, F. Birth prevalence of disorders detectable through newborn screening by race/ethnicity. *Genet Med* **2012**, *14*, 937-945.
97. Therrell, B.L., Jr.; Lloyd-Puryear, M.A.; Eckman, J.R.; Mann, M.Y. Newborn screening for sickle cell diseases in the United States: A review of data spanning 2 decades. *Semin Perinatol* **2015**, *39*, 238-251.
98. Smeltzer, M.P.; Nolan, V.G.; Yu, X.; Nottage, K.A.; Davis, B.A.; Yang, Y.; Wang, W.C.; Gurney, J.G.; Hankins, J.S. Birth Prevalence of Sickle Cell Trait and Sickle Cell Disease in Shelby County, TN. *Pediatr Blood Cancer* **2016**, *63*, 1054-1059.
99. Wang, Y.; Liu, G.; Caggana, M.; Kennedy, J.; Zimmerman, R.; Oyeku, S.O.; Werner, E.M.; Grant, A.M.; Green, N.S.; Grosse, S.D. Mortality of New York children with sickle cell disease identified through newborn screening. *Genet Med* **2015**, *17*, 452-459.
100. Wagner, S.C.; de Castro, S.M.; Gonzalez, T.P.; Santin, A.P.; Zaleski, C.F.; Azevedo, L.A.; Dreau, H.; Henderson, S.; Old, J.; Hutz, M.H. Neonatal screening for hemoglobinopathies: results of a public health system in South Brazil. *Genet Test Mol Biomarkers* **2010**, *14*, 565-569.
101. Lobo, C.L.; Ballas, S.K.; Domingos, A.C.; Moura, P.G.; do Nascimento, E.M.; Cardoso, G.P.; de Carvalho, S.M. Newborn screening program for hemoglobinopathies in Rio de Janeiro, Brazil. *Pediatr Blood Cancer* **2014**, *61*, 34-39.
102. Sabarense, A.P.; Lima, G.O.; Silva, L.M.; Viana, M.B. Survival of children with sickle cell disease in the comprehensive newborn screening programme in Minas Gerais, Brazil. *Paediatr Int Child Health* **2015**, *35*, 329-332.
103. Carlos, A.M.; Souza, R.A.; Souza, B.M.; Pereira Gde, A.; Tostes Junior, S.; Martins, P.R.; Moraes-Souza, H. Hemoglobinopathies in newborns in the southern region of the Triangulo Mineiro, Brazil. Cross-sectional study. *Sao Paulo Med J* **2015**, *133*, 439-444.
104. Silva, W.S.; de Oliveira, R.F.; Ribeiro, S.B.; da Silva, I.B.; de Araujo, E.M.; Baptista, A.F. Screening for Structural Hemoglobin Variants in Bahia, Brazil. *Int J Environ Res Public Health* **2016**, *13*, 225.
105. Eller, R.; da Silva, D.B. Evaluation of a neonatal screening program for sickle-cell disease. *J Pediatr (Rio J)* **2016**, *92*, 409-413.
106. Knight-Madden, J.; Lee, K.; Elana, G.; Elenga, N.; Marcheco-Teruel, B.; Keshi, N.; Etienne-Julan, M.; King, L.; Asnani, M.; Romana, M.; et al. Newborn Screening for Sickle Cell Disease in the Caribbean: An Update of the Present Situation and of the Disease Prevalence. *Int J Neonatal Screen* **2019**, *5*, 5.
107. Alladin, B.A.; Mohamed-Rambaran, P.; Grey, V.; Hunter, A.; Chakraborty, P.; Henderson, M.; Milburn, J.; Tessier, L. Cross-sectional prospective feasibility study of newborn screening for sickle cell anaemia and congenital hypothyroidism in Guyana. *BMJ Open* **2022**, *12*, e046240.
108. Rotz, S.; Arty, G.; Dall'Amico, R.; De Zen, L.; Zanolli, F.; Bodas, P. Prevalence of sickle cell disease, hemoglobin S, and hemoglobin C among Haitian newborns. *Am J Hematol* **2013**, *88*, 827-828.
109. Alvarez, O.A.; Hustace, T.; Voltaire, M.; Mantero, A.; Liberus, U.; Saint Fleur, R. Newborn Screening for Sickle Cell Disease Using Point-of-Care Testing in Low-Income Setting. *Pediatrics* **2019**, *144*.

110. Serjeant, G.R.; Serjeant, B.E.; Mason, K.P.; Gardner, R.; Warren, L.; Gibson, F.; Coombs, M. Newborn screening for sickle cell disease in Jamaica: logistics and experience with umbilical cord samples. *J Community Genet* **2017**, *8*, 17-22.
111. Dokekias, A.E.; Ocko Gokaba, L.T.; Louokdom, J.S.; Ocini, L.N.; Galiba Atipo Tsiba, F.O.; Ondzotto Ibatta, C.I.; Kouandzi, Q.N.; Tamekue, S.T.; Bango, J.C.; Nziengui Mboumba, J.V.; et al. Neonatal Screening for Sickle Cell Disease in Congo. *Anemia* **2022**, *2022*, 9970315.
112. Ojodu, J.; Hulihan, M.M.; Pope, S.N.; Grant, A.M.; Centers for Disease, C.; Prevention. Incidence of sickle cell trait--United States, 2010. *MMWR Morb Mortal Wkly Rep* **2014**, *63*, 1155-1158.
113. Gbadamosi-Akindele, M.; Aurit, S.; Johnson, A.; Nester, A. A State Level Retrospective Analysis of Newborn Screening for Hemoglobinopathies. *Blood* **2019**, *134*, 5806.
114. Telfer, P.; Agodoa, I.; Fox, K.M.; Burke, L.; Mant, T.; Jurek, M.; Tonda, M.; Lehrer-Graiwer, J. Impact of voxelotor (GBT440) on unconjugated bilirubin and jaundice in sickle cell disease. *Hematol Rep* **2018**, *10*, 7643.
115. Bardón Cancho, E.J.; García-Morín, M.; Belendez, C.; Velasco, P.; Beneitez, D.; Ruiz-Llobet, A.; Berrueco, R.; Argiles, B.; Cervera, A.; Salinas, J.A.; et al. Update of the Spanish registry of haemoglobinopathies in children and adults. *Med Clin (Barc)* **2020**, *155*, 95-103.
116. Brousse, V.; Arnaud, C.; Lesprit, E.; Quinet, B.; Odievre, M.H.; Etienne-Julan, M.; Guillaumat, C.; Elana, G.; Belloy, M.; Garnier, N.; et al. Evaluation of outcomes and quality of care in children with sickle cell disease diagnosed by newborn screening: A real-world nation-wide study in France. *J Clin Med* **2019**, *8*, 1594.
117. Desselas, E.; Thuret, I.; Kaguelidou, F.; Benkerrou, M.; de Montalembert, M.; Odievre, M.H.; Lesprit, E.; Rumpler, E.; Fontanet, A.; Pondarre, C.; et al. Mortality in children with sickle cell disease in mainland France from 2000 to 2015. *Haematologica* **2020**, *105*, e440-443.
118. Gualandro, S.F.; Fonseca, G.H.; Yokomizo, I.K.; Gualandro, D.M.; Suganuma, L.M. Cohort study of adult patients with haemoglobin SC disease: clinical characteristics and predictors of mortality. *Br J Haematol* **2015**, *171*, 631-637.
119. Rettenbacher, E.; Zaal, J.; Heijboer, H.; van der Plas, E.M.; Hof, M.; Biemond, B.J.; Fijnvandraat, K.; consortium*, S. Mortality and Causes of Death From Sickle Cell Disease in The Netherlands, 1985-2017. *J Pediatr Hematol Oncol* **2021**, *43*, 258-265.
120. Souza, R.C.; Miranda Neto, P.A.D.; Santos, J.R.N.; Monteiro, S.G.; Goncalves, M.C.; Silva, F.B.; Holanda, R.A.; Santos, J.R.A. Sickle Cell Anaemia Prevalence Among Newborns in the Brazilian Amazon-Savanna Transition Region. *Int J Environ Res Public Health* **2019**, *16*.
